# Structural Properties and Reactive Site Selectivity of Some Transition Metal Complexes of 2,2′(1E,1′E)-(ethane-1,2-diylbis(azan-1-yl-1-ylidene))bis(phenylmethan-1-yl-1-ylidene)dibenzoic Acid: DFT, Conceptual DFT, QTAIM, and MEP Studies

**DOI:** 10.1155/2018/4510648

**Published:** 2018-09-26

**Authors:** Fritzgerald Kogge Bine, Nyiang Kennet Nkungli, Tasheh Stanley Numbonui, Julius Numbonui Ghogomu

**Affiliations:** Department of Chemistry, Faculty of Science, Research Unit of Noxious Chemistry and Environmental Engineering, University of Dschang, P.O. Box 67, Dschang, Cameroon

## Abstract

Herein is presented a density functional theory (DFT) study of reactivity and structural properties of transition metal complexes of the Schiff base ligand 2,2′(1E,1′E)-(ethane-1,2-diylbis(azan-1-yl-1-ylidene))bis(phenylmethan-1-yl-1-ylidene)dibenzoic acid (hereafter denoted EDA2BB) with Cu(II), Mn(II), Ni(II), and Co(II). The quantum theory of atoms-in-molecules (QTAIM), conceptual DFT, natural population analysis (NPA), and molecular electrostatic potential (MEP) methods have been used. Results have revealed a distorted octahedral geometry around the central metal ion in each gas phase complex. In the DMSO solvent, a general axial elongation of metal-oxygen bonds involving ancillary water ligands has been observed, suggestive of loosely bound water molecules to the central metal ion that may be acting as solvent molecules. Weak, medium, and strong intramolecular hydrogen bonds along with hydrogen-hydrogen and van der Waals interactions have been elucidated in the complexes investigated via geometric and QTAIM analyses. From the chemical hardness values, the complex [Co(EDA2BB)(OH_2_)_2_] is the hardest, while [Cu(EDA2BB)(OH_2_)_2_] is the softest. Based on the global electrophilicity index, the complexes [Ni(EDA2BB)(OH_2_)_2_] and [Cu(EDA2BB)(OH_2_)_2_] are the strongest and weakest electrophiles, respectively, among the complexes studied. In conclusion, the reactivity of the complexes is improved vis-à-vis the ligand, and stable geometries of the complexes are identified, alongside their prominent electrophilic and nucleophilic sites.

## 1. Introduction

The development of resistance to currently used pharmaceutical drugs by pathogens is a direct consequence of drug abuse, thereby necessitating the search for new and more potent chemical agents with increased therapeutic properties against disease-causing microorganisms [1]. This also explains the appearance of superbugs which are drug-resistant strains of microorganisms that only respond to the most powerful antibiotics, or in some cases, to no antibiotics. In the search for new drugs, transition metals have been identified to possess unique biological activities on pathogenic organisms and thus provide a pathway for the synthesis of new drugs [2].

Transition metal complexes derived from salen-type ligands are of particular interest in medicinal chemistry for the development of antimicrobial agents. Indeed, Schiff base compounds and their metal complexes are outstanding in the domain of metal-based drugs. They have been extensively investigated in this perspective firstly because of their wide range of applications in medicine [3]. Secondly, much interest has been dedicated to their study because of their synthetic flexibility, selectivity, and sensitivity towards the central metal atom/ion. Interestingly, the structural similarities of Schiff base reagents (Schiff base ligands and their metal complexes) to biomolecules facilitate elucidation of the biological mechanisms of transformation and racemization reactions [4].

Recently, Al-Shemary and Zaidan [5] reported the successful synthesis and characterization of a new tetradented Schiff base ligand known as 2,2′(1E,1′E)-(ethane-1,2-diylbis(azan-1-yl-1-ylidene))bis(phenylmethan-1-yl-1-ylidene)dibenzoic acid (EDA2BB) and its Cu^II^, Ni^II^, Mn^II^, Co^II^, and Hg^II^ complexes. Although EDA2BB and its metal complexes showed good antibacterial activities on some selected targets, a comprehensive analysis of their geometric and structural properties, as well as their chemical reactivity and stability, has not been carried out till date, to the best of our knowledge. To address these issues, we embarked on a quantum chemical study of the structural properties and reactive site selectivity of EDA2BB and its metal complexes. The success of the molecular docking of a drug into its receptor site, which usually involves an interplay of precise molecular interactions, depends greatly on the geometry, conformation, and electronic properties of the two molecules [6]. Therefore, our current theoretical study on the transition metal complexes of EDA2BB is a particularly important prerequisite for any future docking investigations using these molecules as “Ligands”.

**Figure 1:**
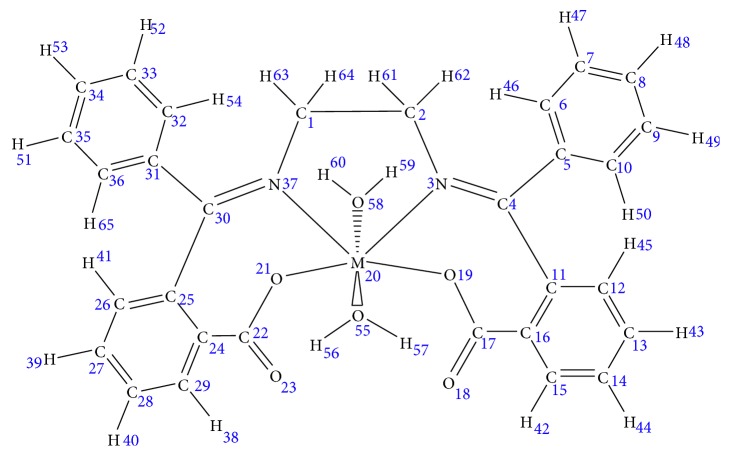
Input geometries with formula [**M**(**N**_2_ **O**_4_ **C**_30_ **H**_22_)(**O** **H**_2_)_2_], where **M**_20_ = **Cu(II)**, **Ni(II)**, **Mn(II)** and **Co(II)**.

The main objective of the present study was to perform a geometric and structural analysis on some transition metal complexes of the ligand EDA2BB, comprising [Cu(EDA2BB)(OH_2_)_2_] (**A**), [Ni(EDA2BB)(OH_2_)_2_] (**B**), [Mn(EDA2BB)(OH_2_)_2_] (**C**), and [Co(EDA2BB)(OH_2_)_2_] (**D**), using the density functional theory (DFT) method and the quantum theory of atoms-in-molecules (QTAIM) analysis. The general molecular structure of these complexes is depicted in Figure 1. Thereafter, the chemical reactivity and reactive site selectivity of these complexes were elucidated via conceptual DFT, molecular electrostatic potential (MEP), and natural population analysis (NPA) studies. The choice of the DFT over the Hatree–Fock and semiempirical methods was motivated by its good compromise between cost (with respect to computational time and hardware requirements) and accuracy [7]. Today, parallel with the impressive development of computational hardware, the quantum chemical software required to perform DFT calculations has progressed to the state where calculations can be performed with high efficiency and in a user-friendly manner [8].

## 2. Computational Details

All theoretical calculations were carried out with the ORCA 3.0.3 computational package [9]. The input files were prepared using Avogadro 1.1.1 [10]. Geometry optimization and frequency calculations were performed using the DFT method in conjunction with the def2-TZVP(-f) Ahlrichs basis set [11]. The GGA functional BP86 [12, 13] was chosen for these calculations because it provides excellent geometries and vibrational frequencies [8]. To speed-up the geometry optimization and frequency calculations, but with marginal loss in accuracy, the resolution-of-the-identity (RI-J) approximation [14] was used in conjunction with appropriate auxiliary basis sets. Solvent effects were simulated using the conductor-like screening model (COSMO) [15] that is well-integrated into the ORCA program system. Dimethylsulfoxide (DMSO) was used as a solvent in this work in order to employ the same solvent as that used by Al-Shemary and Zaiden [5] in their experimental studies. Furthermore, long-range dispersion interactions were incorporated via Grimme's atom-pairwise dispersion correction using the Becke–Johnson damping scheme (D3BJ) [16, 17], since the pure exchange-correlation functionals fail to properly account for such interactions.

The geometries of the Cu(II), Co(II), and Mn(II) complexes of EDA2BB were optimized using the Unrestricted Kohn–Sham (UKS) formalism, while the Ni(II) complex was optimized using the Restricted Kohn–Sham (RKS) formalism. No constraints on symmetry, bond lengths, bond angles, or dihedral angles were applied in the geometry optimization calculations. In order to be sure that we obtained reliable and stable structures (minima on the PES), vibrational frequencies were calculated for all optimized structures, and their analyses showed no imaginary frequencies.

In the implementation of conceptual DFT for the evaluation of ionization energies and electron affinities, the hybrid *Meta*-GGA functional, M06 [18] was used for single point energy calculations based on the BP86-optimized geometries. This functional was chosen because it incorporates an inbuilt dispersion correction and performs well for energy calculations [18]. The M06 functional was employed along with the basis set ma-def2-TZVP for the metal ions and ma-def2-SVP for every other element. To speed-up these calculations, the RI-J approximation was combined with the chain-of-spheres (COSX) approximation, giving rise to the RIJCOSX approximation [19]. Throughout our ORCA calculations, numerical quadrature grids of at least 4 were used. NPA calculations were performed on the geometries optimized at BP86/def2-TZVP(-f) level, with the aid of the JANPA computational package [20].

## 3. Results and Discussion

### 3.1. Structural and Geometric Analysis

**Figure 2:**
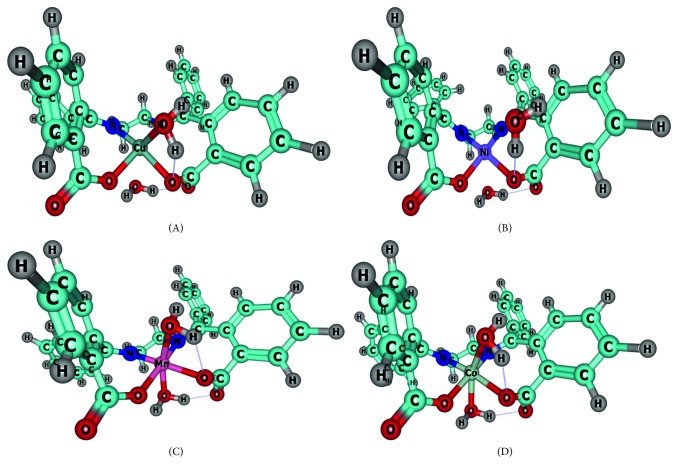
The optimized geometries of [**C** **u**(**E** **D** **A**2**B** **B**)(**O** **H**_2_)_2_] (**A**), [**N** **i**(**E** **D** **A**2**B** **B**)(**O** **H**_2_)_2_] (**B**), [**M** **n**(**E** **D** **A**2**B** **B**)(**O** **H**_2_)_2_] (**C**), and [**C** **o**(**E** **D** **A**2**B** **B**)(**O** **H**_2_)_2_] (**D**) at the RI-BP86-D3(BJ)/def2-TZVP(-f) level of theory in gas phase.

The input geometries to ORCA consisted of octahedral complexes, each containing one EDA2BB ligand and two water molecules serving as ancillary ligands to a transition metal(II) ion, as previously suggested by Al-Shemary and Zaiden [5]. Selected geometric parameters (bond lengths, bond angles, and dihedral or torsional angles) of the complexes investigated herein are listed in Table 1. The optimized geometries of the complexes are shown in Figure 2, as visualized using the Chemcraft 1.8 visualization software [21].

It can be seen from Table 1 that nearly all metal-ligand (M-L) bond lengths calculated in both gas phase and DMSO fall within the range of typical M-L bond distances, 1.849–2.087 au [22]. The relatively large values of the M-O_55_ and M-O_58_ bond lengths in the complexes currently investigated, 2.888–3.531 au suggest that the water ligands are loosely bound to the central metal ions. A general axial elongation of the M-O bonds involving the ancillary water ligands is observed in DMSO, suggesting that the water ligands are much more loosely bound to the central metal ions in the solvent phase and may instead be acting as solvent molecules. This is probably caused by some intermolecular interactions between the water molecules and the DMSO solvent molecules. Moreover, ligand substitution reactions in which the water ligands are replaced by DMSO molecules may occur.

The M-L bond angles indicate distorted octahedral geometries around the central metal ions in the complexes studied. These distortions are probably imposed by the chelate rings. If the complexes under investigation were to be perfectly octahedral, all of the dihedral angles listed in Table 1 will be 0^o^ each, except the angles O_21_-M-N_37_-C_1_ and O_19_-M-N_3_-C_2_ which will be 180^o^ instead. Upon inspection of Table 1, however, the dihedral angles O_21_-M-N_37_-C_1_ and O_19_-M-N_3_-C_2_ are found to have values in the range −117.2° to −135.4° and 129.2° to 164.1° respectively, which deviate significantly from 180°. Moreover, the values of the other torsional angles show a maximum deviation of 103.5° from 0°. These results confirm that the optimized structures of the complexes investigated are distorted octahedrons.

The intramolecular hydrogen bond (HB) parameters in the complexes studied, which were determined based on the categorization of HBs by Jeffrey [23], are also listed in Table 1. Geometric cutoff limits for HB interactions (herein denoted X–H⋯A, where X–H is the donor and A is the acceptor) were employed in identifying potential hydrogen bonding cases. The cut-off limits used in this paper are H⋯A distances up to 3.2 Å and X–H⋯A angles ≥90°. From Table 1, it can be seen that the HB lengths in the complexes optimized in the gas phase are shorter than those in the complexes optimized in the solvent phase, implying that the HBs in the gas phase complexes could be stronger than those in the solvent phase counterparts. It is worth noting that the HB donors of all the intramolecular HBs described in Table 1 are the water ligands. Hence, the elongation of the HBs in the solvent phase can be attributed to some intermolecular HB interactions between the water Ligands and the polar solvent (DMSO) molecules, which probably act as the HB acceptors. Based on the aforementioned geometric cut-off limits for HB interactions, a complete disappearance of the HB interactions ongoing from the gas phase to the solvent phase is observed in some complexes, notably the Co(II), Mn(II), and Cu(II) complexes.

### 3.2. Infrared Spectroscopy

In the realm of theoretical chemistry, frequency calculations not only characterize stationary points (as minima, transition states, etc.), but are also used to predict the IR spectra of molecules. In this regard, the IR spectra of the molecules currently investigated have been calculated, and some pertinent vibrational frequencies of these molecules are listed in Table 2. Also listed in Table 2 are the experimentally determined vibrational frequencies obtained from Al-Shemary and Zaiden [5]. The theoretical and experimental IR frequencies are compared in this work as a means of determining the credibility of the level of theory used in geometry optimization. The computed IR spectra have also been used to provide some insights into geometric structures of the molecules studied.

Usually, calculated IR frequencies are larger than the corresponding experimental values. To obtain a good agreement between the experimental and the calculated wavenumbers, the latter are usually corrected with appropriate scale or correction factors [24]. In the present study, the scale factor 0.9953, which is suitable for correcting wavenumber computed at the BP86/def2-TZVP level of theory [25], has been used.

To determine the validity of the calculated frequencies with respect to the experimental values, the correlation Equation (1) was established:

(1) $$\begin{array}{c} \nu_{\text{calc}.}=0.980\nu_{\exp.}+27.20, \end{array}$$

where *ν*_calc._ and *ν*_exp._ represent the calculated (scaled) and the experimental IR frequencies, respectively. The correlation coefficient (*R* ^2^=0.999) obtained between the theoretical and experimental FT-IR frequencies shows a good linear relationship between these values, thus affirming the suitability of the level of theory used for geometry optimization. In particular, a good agreement is found to exist between the O-H vibrational frequencies of the water ligands and the experimental values. Good agreements between theoretical and experimental frequencies are also found to exist for the aliphatic C-H, aromatic C-H, the azomethine C=N, and M-N vibrations.

The IR spectrum of the ligand is found to be more or less modified by metal coordination. Upon complexation, the emergence of O-H bands due to the water ligands is observed in the IR spectrum of EDA2BB in the region 3300–3600 cm^−1^. Furthermore, IR bands due to the metal-ligand (Cu-O and Cu-N) vibrations also appeared in the spectrum of EDA2BB in the range 350–700 cm^−1^. Moreover, the strengthening of the carbonyl and the imine IR bands of EDA2BB was observed after complexation, which confirms that the ligand coordinates to the central metal ions via the carbonyl oxygen and the azomethine nitrogen. The disappearance of the IR bands corresponding to the O-H vibrations of EDA2BB indicates the loss of its carboxylic protons upon complexation with the metal ions.

### 3.3. QTAIM Analyses on the Complexes Investigated

**Figure 3:**
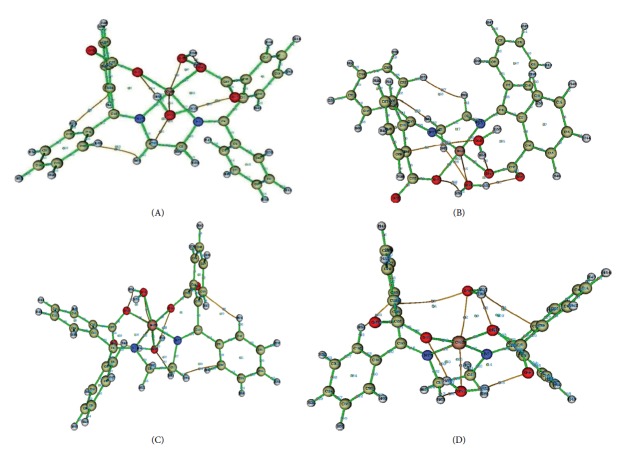
Molecular graphs of EDA2BB complexes computed based on their gas phase optimized geometries at the BP86-D3(BJ)/def2-TZVP(-f) level of theory. The BCPs are the small red spheres around the centre of the bond paths. The bond paths are the brown or green colored lines.

To ascertain the existence of HBs alongside other interactions such as van der Waals interactions in the complexes studied, Bader's quantum theory of atoms-in-molecules (QTAIM) [26] approach was employed. QTAIM analyses on the complexes were carried out using the Multiwfn 3.3.9 software [27]. It is worthy of note that the nature of the chemical bonds in transition metal complexes remains an area of ongoing research [28]. Critical points were searched via topology analysis of the electron density. In topology analysis language, the points at which the gradient of the electron density goes to zero (except at infinity) are known as critical points (CPs). In this paper, bond CPs (BCP) were of most interest. The Pointcaré–Hopf relationship was satisfied each time a critical point search was performed, indicating that all CPs may have been found.  Figure 3 shows the molecular graphs of the EDA2BB-metal complexes studied, based on their gas phase optimized geometries at RI-BP86-D3(BJ)/def2-TZVP(-f) level of theory.

Bader's QTAIM analysis [26] has become the first choice tool used by quantum chemists to analyze the nature and strengths of bonding interactions. In the present study, topological analyses of the electron density and its Laplacian have been performed at bond critical points. Large *ρ*(*r*) values and ∇^2^ *ρ*(*r*) < 0 indicate polar and nonpolar covalent bonding interactions, whereas small *ρ*(*r*) values and ∇^2^ *ρ*(*r*) > 0 indicate closed-shell interactions [29]. Generally, *ρ*(*r*) is greater than 0.20 au for covalent bonding interactions and less than 0.10 au for closed-shell interactions [29]. The values of ∇^2^ *ρ*(*r*) and –*G*(*r*)/*V*(*r*) were also used to characterize the bonding interactions in the complexes of EDA2BB investigated. Generally, *G*(*r*) is the kinetic energy density at the BCP (always positive), and *V*(*r*) is the potential energy density at the BCP (always negative) [30]. When ∇^2^ *ρ*(*r*) > 0 and –*G*(*r*)/V (*r*) > 1, the interactions are noncovalent, whereas when ∇^2^ *ρ*(*r*) > 0 and 0.5 < −*G*(*r*)/*V*(*r*) < 1, the interactions are partially or partly covalent [29].

In the context of QTAIM analysis, Popelier [31] developed some useful criteria for characterizing HBs, which have been exploited in this work. According to Popelier, the formation an HB depends on the electron density and its Laplacian at the BCP, which should lie in the range of 0.002–0.040 au and 0.024–0.139 au, respectively. Espinosa [32] formulated an equation of the form *E*_int_ = 0.5*V*(*r*), which is useful for estimating weak interatomic interaction energies, particularly HB energies. In this equation, *V*(*r*) is the potential energy density.

The computed parameters at some BCPs in the complexes studied are listed in Table 3. It is clear from this table that all ∇^2^ *ρ*(*r*) values are positive, except that for the interaction H_64_⋯C_36_ in C (−0.584 au) which is negative. Furthermore, the *ρ*(*r*) value for this interaction is greater than 0.20 au, but less than 0.10 au for all the other interactions in this complex. These results show that the H_64_⋯C_36_ in C is the only covalent interaction among the interactions list in Table 3. This conclusion is further supported by the large interaction energy (−80.949 kcal/mol) for the H_64_⋯C_36_ interaction, as compared to the interaction energies of the other interactions, which are in the range −0.314 to −67.143 kcal/mol.

Based on the interaction energies listed in Table 3 and on Jeffrey's classification of HBs [23], the conventional HBs in the complexes under investigation have been classified as weak (0–4 kcal/mol), medium (4–14 kcal/mol), or strong (14–40 kcal/mol). For the complex C, the HBs H_63_⋯O_58_, H_56_⋯O_19_, and H_59_⋯O_18_ are weak, medium, and strong, respectively. For the complexes A and B, the HBs H_63_⋯O_58_ and O_21_⋯H_60_ are weak, whereas H_59_⋯O_18_ and H_56_⋯O_19_ are medium. For D, the HB H_63_⋯O_58_ is weak, while H_59_⋯O_18_, H_56_⋯O_19_, and O_21_⋯H_60_ are medium. Additionally, hydrogen-hydrogen (H–H) interactions have been found to exist in B (H_54_⋯H_61_) and D (H_54_⋯H_41_). Furthermore, some interactions of the form C⋯H in the complexes studied have been identified as intramolecular van der Waal's interactions, since their interaction energies (−0.914 to −2.510 kcal/mol) are lower than the interaction energies of virtually all of the hydrogen bonds identified.

### 3.4. Electronic Properties

#### 3.4.1. Natural Population Analysis (NPA)

To determine the charge redistribution and the extent of metal-ligand charge transfer in the complexes investigated, NPA charges (also known as natural charges) were computed at BP86-D3(BJ)/def2-TZVP(-f) level of theory in gas phase. The natural charges on selected atoms of the investigated compounds are listed in Table 4.

It is clear from Table 4 that the formal charge (+2) on each central metal ion decreases upon complexation with EDA2BB. The formal charge on Ni^2+^, Cu^2+^, Mn^2+^, and Co^2+^ decreased to +0.9308, +1.2323, +0.9438, and +1.0003, respectively. From these results, it is obvious that electron transfer from the ligand to each central metal ion occurred during complex formation. Clearly, these metal-ligand charge transfers are substantial in the Ni(II) and Mn(II) complexes, and less significant in the Cu(II) and Co(II) complexes. Note that in metal complexes, the more the reduction in polarity of the central metal, the more delocalized are π-electrons over the chelate ring, and this enhances the lipophilicity of the complexes [33]. This increased lipophilicity in turn enhances the penetration of the complexes into lipid membranes and the blocking of the metal binding sites in some enzymes found in microorganisms [33]. From this viewpoint, it can be affirmed from Table 4 that the Ni(II) and Mn(II) complexes of EDA2BB are potentially highly lipophilic toward the penetration of the cell membranes of microorganisms, while the Cu(II) and Co(II) complexes are least penetrating.

#### 3.4.2. Global Reactivity Descriptors (GRDs)

The stability and chemical reactivity of the complexes investigated were studied via conceptual density functional theory (CDFT), using the global reactivity descriptors. The energies necessary for the computation of these global reactivity descriptors were obtained from single-point calculations at M06/ma-def2-TZVP level of theory for the metal ions and at M06/ma-def2-SVP level of theory for every other element, based on the optimized geometries of the complexes. The Minnesota functional, M06 has been chosen here because it has been parameterized to provide accurate energies of transition metal complexes [18].

Within the framework of CDFT, global reactivity descriptors such as Chemical potential (*μ*), Electronegativity index (*χ*), Chemical hardness (*η*), and Chemical softness (*S*) have been defined. A detailed description of these reactivity descriptors can be found in our previous publication [34]. Note that all of these reactivity parameters are related to the response of chemical systems to changes in either their number of electrons or changes in the external potential, or both, which provide information about their reactivity [35]. To better describe the molecular reactivity of the complexes studied, our discussions have been based on the molecular electron density theory (MEDT) put forth by Domingo and coworkers [35]. This theory states that “while the electron density distribution at the ground-state is responsible for physical and chemical molecular properties, the capability for changes in electron density, and not molecular orbital interactions, is responsible for molecular reactivity”.

The electronic chemical potential, *μ*, is associated with the feasibility of a system to exchange electron density with the environment at the ground state. The electronegativity, *χ*, is a measure of the resistance to electron density loss. The chemical hardness, *η*, is thought of as the resistance of a molecule to electron density exchange with the environment. On the other hand, the chemical softness, *S*, is the inverse of the chemical hardness, *η*. The values of the global reactivity descriptors computed in this work are reported in Table 5.

A careful inspection of Table 5 revealed that *μ* varies in the order **B** < **D** < **C** < **A**, which indicates that the Cu(II) complex is the most reactive with respect to electron-donation, while the Ni(II) complex is the most reactive in terms of electron acquisition. On the other hand, *χ* varies thus **A** < **C** < **D** < **B**, which confirms the Ni(II) complex as the most susceptible to electron gain, while the Cu(II) complex is the most resistant to electron density gain. It is clear from the ranking of *η* values **A** < **C** < **B** < **D**, that of all the complexes studied, the Co(II) complex is the hardest (chemically) and the Cu(II) complex is the most chemically soft. The global electrophilicity index, *ω*, a measure of the energy stabilization of a molecule when it acquires an additional amount of electron density, is one of the most important reactivity indices. Generally, electrophiles are characterized by high *ω* values [35]. On this basis, the strongest electrophile among the molecules studied is the Ni(II) complex, while the Cu(II) complex is the weakest electrophile.

### 3.5. Molecular Electrostatic Potential (MEP) Surfaces

Molecular Electrostatic Potential, *Ф*(*r*), has been widely used for predicting the sites that are susceptible to nucleophilic, electrophilic, and free radical attacks on molecular species, as well as molecular docking modes for quite a long time. *Ф*(*r*) is calculated using

(2) $$\begin{array}{c} Ф\left(r\right)=\int \frac{\rho_{\text{total}}\left(r\right)}{\left|r-r^{\prime }\right|}dr, \end{array}$$

where *ρ*_total_ represents both the nuclear and the electronic charge density. The integration is over the molecular volume, and *r*′ represents the atomic positions relative to the same origin. The integration includes the atoms of only one molecule and therefore does not directly include the effects of charge distributions of the neighboring molecules [36].

**Figure 4:**
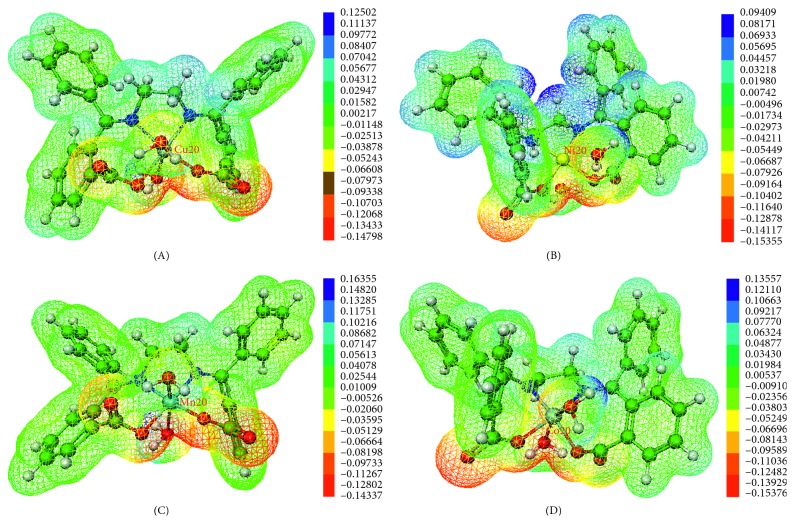
Molecular electrostatic potential (MEP) surfaces of EDA2BB complexes, mapped onto electron density isosurfaces of value 0.01 au, using Molekel 4.3.

To elucidate the sites for molecular reactivity in the investigated complexes in the gas phase, their MEP maps were computed at RIJCOSX-M06/def2-TZVP level of theory in the gas phase, and visualized (as shown in Figure 4) via the Molekel 4.3 graphical user interface [37]. In this figure, the color code red to yellow indicates regions of low electrostatic potential and is related to electrophilic reactivity, while blue to green indicates regions of high electrostatic potential related to nucleophilic reactivity sites. It should be noted that the values reported in the legend are in au.

The most negative regions of the MEP maps are associated with the oxygen atoms of both the water ligands and the Schiff base ligand. In particular, the oxygen atoms O_23_ and O_18_ in each complex (with electrostatic potentials ranging from −0.1537 au in D to −0.14337 au in B) are found to be the most suitable sites for electrophilic attack. The C_1_⋯H_63_, C_1_⋯H_64_, C_2_⋯H_61_, and C_2_⋯H_62_ bonds, as well as the hydrogen atoms (H_57_, H_60_, H_59_, and H_56_) of the water ligands in all of the complexes are seemingly the most probable sites for nucleophilic attack. This is shown by the relatively high electrostatic potentials in the neighborhood of these atoms, which range from 0.16355 au in C to 0.09409 au in B. It is worthy to note that these hydrogen atoms are responsible for both intramolecular and intermolecular HB formation by the complexes studied. Such intermolecular HBs could be formed with electronegative atoms in protein molecules present in the cell walls of microorganisms, resulting in interference with the normal cell processes [5].

## 4. Conclusion

Literature holds that some synthetic first-row transition metal complexes of the tetradentate Schiff base ligand 2,2′(1E,1′E)-(ethane-1,2-diylbis(azan-1-yl-1-ylidene))bis(phenylmethan-1-yl-1-ylidene)dibenzoic acid (herein denoted EDA2BB) and two water molecules acting as ancillary ligands possess interesting antimicrobial effects [5]. This work addresses a detailed analysis of their structural properties and chemical reactivity via theoretical studies on some transition metal complexes of EDA2BB: *[Cu(EDA2BB)(OH*_*2*_)_*2*_ *]*, *[Ni(EDA2BB)(OH*_*2*_)_*2*_ *]*, *[Mn(EDA2BB)(OH*_*2*_)_*2*_ *]*, and *[Co(EDA2BB)(OH*_*2*_)_*2*_ *]* via the DFT method. The DFT studies have been performed using the exchange-correlation functionals BP86 and M06. These functionals were employed together with the basis sets ma-def2-SVP, ma-def2-TZVP, and def2-TZVP(-f). Calculations were performed in gas phase, as well as in DMSO in order to simulate solvent effect. The quantum theory of atoms-in-molecules (QTAIM), conceptual DFT, natural population analysis (NPA), and molecular electrostatic potential (MEP) methods were also employed. The results obtained have revealed a distorted octahedral geometry around the central metal ion in each complex studied in gas phase. In DMSO solvent, a general axial elongation of the metal-oxygen bonds involving the water ligands has been observed, suggesting that the water ligands are loosely bound to the central metal ions and might be acting as solvent molecules. Weak, medium, and strong intramolecular hydrogen bonds, along with hydrogen-hydrogen and van der Waals interactions, have been elucidated in the complexes, via geometric and QTAIM analyses. From the NPA charges on the central metal ions, it was found that electron transfer from the ligand EDA2BB to the central metal ions occurred during complex formation. Clearly, these metal-ligand charge transfers are substantial in the Ni(II) and Mn(II) complexes and less significant in the Cu(II) and Co(II) complexes. From the chemical hardness values, the complex *[Co(EDA2BB)(OH*_*2*_)_*2*_ *]* is the hardest, while *[Cu(EDA2BB)(OH*_*2*_)_*2*_ *]* is the softest. Based on the global electrophilicity index, the strongest electrophile among the complexes studied is *[Ni(EDA2BB)(OH*_*2*_)_*2*_ *]*, whereas *[Cu(EDA2BB)(OH*_*2*_)_*2*_ *]* is the weakest electrophile. The most negative regions of the MEP maps are associated with the oxygen atoms of both the water ligands and the Schiff base ligand. In particular, the oxygen atoms O_23_ and O_18_ in each complex are found to be the most suitable sites for electrophilic attack. The C_1_⋯H_63_, C_1_⋯H_64_, C_2_⋯H_61_, and C_2_⋯H_62_ bonds, as well as the hydrogen atoms (H_57_, H_60_, H_59_, and H_56_) of the water ligands in all of the complexes studied are seemingly the most probable sites for nucleophilic attack. In a nutshell, therefore, we have shown that the activities of the complexes are improved vis-à-vis the ligands, and stable geometries of the complexes have been identified, alongside their prominent electrophilic and nucleophilic sites.

## Figures and Tables

**Table 1:** Selected optimized geometric parameters of the complexes investigated in gas phase and DMSO.

Geometric parameters	Gas phase				DMSO			
	**A**	**B**	**C**	**D**	**A**	**B**	**C**	**D**
Bond lengths (Å)								
**M**_20_-**N**_37_	1.973	1.849	1.912	1.864	1.972	1.845	1.911	1.879
**M**_20_-**N**_3_	2.087	1.913	1.997	1.935	2.066	1.895	1.981	1.900
**O**_21_-**M**_20_	1.965	1.857	1.943	1.918	2.022	1.877	2.019	1.923
**O**_19_-**M**_20_	2.015	1.890	2.021	1.981	1.975	1.872	2.045	1.917
**O**_55_-**M**_20_	2.452	3.268	2.203	2.346	2.288	3.336	2.103	2.203
**O**_58_-**M**_20_	2.846	2.888	2.138	2.360	3.243	3.531	2.168	3.463

Bond angles (°)								
**O**_21_-**M**_20_-**N**_37_	89.4	91.0	90.5	89.9	87.3	90.9	89.2	91.6
**N**_3_-**M**_20_-**O**_19_	98.2	99.3	95.2	99.9	98.1	99.8	97.8	93.9
**N**_3_-**M**_20_-**N**_37_	81.3	82.7	84.2	84.5	81.5	83.4	84.7	83.3
**O**_58_-**M**_20_-**O**_19_	77.1	78.8	87.2	82.5	65.5	58.1	83.3	55.0
**O**_55_-**M**_20_-**O**_19_	74.8	63.4	77.3	75.4	95.0	61.0	87.5	99.8

Dihedral angles (°)								
**O**_21_-**M**_20_-**N**_37_-**C**_30_	45.5	66.0	40.6	49.3	52.8	67.8	42.7	61.3
**O**_19_-**M**_20_-**N**_3_-**C**_4_	−41.3	−43.9	−26.6	−33.0	−50.3	−39.9	−32.9	−54.7
**O**_21_-**M**_20_-**N**_37_-**C**_1_	−118.4	−119.7	−135.4	−127.7	−117.2	−119.7	−135.0	−119.3
**O**_19_-**M**_20_-**N**_3_-**C**_2_	147.3	144.0	164.1	153.7	140.2	147.4	159.3	129.2
**M**_20_-**O**_21_-**C**_22_-**C**_24_	−4.9	−11.7	−5.6	−9.8	−9.1	−12.6	−10.3	−25.2
**M**_20_-**O**_19_-**C**_17_-**C**_16_	90.1	90.1	103.5	95.1	78.8	78.0	97.1	27.0
**C**_1_-**N**_37_-**C**_30_-**C**_31_	11.2	−1.4	23.9	18.8	11.1	−1.3	23.1	−6.5
**C**_2_-**N**_3_-**C**_4_-**C**_5_	2.5	8.5	6.1	3.3	3.0	6.0	5.3	−0.7

Hydrogen bond lengths (Å)								
**H**_56_⋯**O**_19_	1.917	1.996	1.978	1.879	—	2.928	3.103	—
**H**_59_⋯**O**_18_	1.920	1.998	1.673	1.833	2.045	2.052	1.743	—
**H**_60_⋯**O**_21_	2.105	2.338	2.004	2.010	2.024	2.061	2.158	1.960
**O**_19_⋯**H**_59_	—	—	—	—	—	—	—	1.960
**N**_3_⋯**H**_57_	3.317	—	2.655	2.962	—	—	—	—

Hydrongen bond angles (°)								
**O**_55_-**H**_56_-**O**_19_	138.1	164.1	122.1	133.5	—	99.8	—	—
**O**_18_-**H**_59_-**O**_58_	160.9	162.6	157.8	155.5	173.6	175.6	156.6	
**O**_58_-**H**_60_-**O**_21_	138.0	135.8	114.1	123.0	163.2	166.2	110.2	146.4
**N**_3_-**H**_57_-**O**_55_	90.1	—	97.8	91.2	—	—	—	—
**O**_19_-**H**_59_-**O**_58_	—	—	—	—	—	—	—	146.8

M = Cu(II), Ni(II), Mn(II), and Co(II). For atom numbering, refer to Figure 1.

**Table 2:** Calculated IR frequencies (scaled) at RI-BP86/def2-TZVP level, along with the corresponding experimental IR frequencies for EDA2BB and its metal(II) complexes.

Vibrational assignment	**A**		**B**		**C**		**D**		**EDA2BB**	
	Calc.	Exp.	Calc.	Exp.	Calc.	Exp.	Calc.	Exp.	Calc.	Exp.
*v*(OH)	3386	3367	3451.7	3423	3392	3321	3363	3482	3113	2520–3337
*v*(C-H)_aroma._	3080	3042	3081	3082	3075	3026	3079	3018	3081	3020
*v*(C-H)_aliph._	2925	2910	2960	2904	2966	2993	2972	2964	2962	2993
*v*(HC=N)_imine_	1601	1609	1598	1614	1582	1604	1570	1612	1611	1627
*v*(C=O)_asym_	1611	1533	1628	1519	1644	1529	1636	1529	1649	—
*v*(M-O)	368	432	387	412	401	418	413	421	—	—
*v*(M-N)	512	520	506	493	493	499	477	489	—	—

Calc. stands for the calculated and scaled IR wavenumbers. Exp. represents the experimentally determined counterparts.

**Table 3:** Bonding interactions and values of the electron density [*ρ*(*r*)] and its Laplacian [∇^2^ *ρ*(*r*)], kinetic energy density [*G*(*r*)], potential energy density [*V*(*r*)], and interatomic interaction energy (E_int_) for the noncovalent and covalent bonding interactions in the complexes studied.

Complex	Bonding interaction	*ρ*(*r*)	∇^2^ *ρ*(*r*)	*G*(*r*)	*V*(*r*)	−*G*(*r*)/*V*(*r*)	E_int_ (kcal/mol)
**A**	H_54_⋯C_26_	0.011	0.036	0.008	−0.006	1.239	−1.883
	H_64_⋯C_36_	0.012	0.043	0.009	−0.008	1.213	−2.510
	H_63_⋯O_58_	0.018	0.061	0.013	−0.011	1.19	−3.451
	H_56_⋯O_19_	0.030	0.102	0.025	−0.025	1.009	−7.844
	H_59_⋯O_18_	0.028	0.087	0.021	−0.021	1.022	−6.589
	O_21_⋯H_60_	0.017	0.071	0.015	−0.012	1.23	−3.765
	Cu_20_⋯O_55_	0.030	0.119	0.030	−0.031	0.983	−9.726
	Cu_20_⋯O_58_	0.015	0.044	0.011	−0.011	0.997	−3.451
	Cu_20_⋯N_3_	0.072	0.267	0.084	−0.101	0.831	−31.689
	Cu_20_⋯N_37_	0.094	0.346	0.116	−0.144	0.8	−45.181
	Cu_20_⋯O_19_	0.074	0.324	0.098	−0.116	0.849	−36.395
	Cu_20_⋯O_21_	0.086	0.364	0.114	−0.136	0.834	−42.671

**B**	H_54_⋯C_26_	0.011	0.036	0.008	−0.006	1.239	−1.883
	H_64_⋯C_36_	0.012	0.043	0.009	−0.008	1.213	−2.510
	H_54_⋯H_61_	0.006	0.001	0.004	−0.003	1.295	−0.941
	H_64_⋯C_31_	0.014	0.046	0.010	−0.008	1.181	−2.510
	H_63_⋯O_58_	0.013	0.042	0.009	−0.007	1.262	−2.196
	H_56_⋯O_19_	0.024	0.078	0.018	−0.016	1.092	−5.020
	H_59_⋯O_18_	0.023	0.076	0.017	−0.015	1.111	−4.706
	O_21_⋯H_60_	0.011	0.044	0.009	−0.007	1.327	−2.196
	C_24_⋯O_55_	0.003	0.011	0.002	−0.001	1.611	−0.314
	Ni_20_⋯O_58_	0.015	0.043	0.011	−0.012	0.962	−3.765
	Ni_20_⋯N_3_	0.106	0.450	0.143	−0.174	0.823	−54.593
	Ni_20_⋯N_37_	0.125	0.520	0.172	−0.214	0.803	−67.143
	Ni_20_⋯O_19_	0.097	0.494	0.147	−0.171	0.862	−53.652

**C**	Ni_20_⋯O_21_	0.111	0.519	0.160	−0.190	0.842	−59.613
	H_54_⋯C_26_	0.013	0.041	0.009	−0.007	1.200	−2.196
	H_64_⋯C_36_	0.246	−0.584	0.056	−0.258	0.217	−80.949
	H_63_⋯O_58_	0.012	0.050	0.010	−0.008	1.253	−2.510
	H_56_⋯O_19_	0.027	0.108	0.025	−0.023	1.084	−7.216
	H_59_⋯O_18_	0.051	0.113	0.039	−0.050	0.779	−15.688
	O_21_⋯O_58_	0.026	0.121	0.027	−0.024	1.129	−7.530
	Mn_20_⋯O_55_	0.048	0.216	0.059	−0.064	0.921	−20.080
	Mn_20_⋯O_58_	0.056	0.276	0.075	−0.081	0.929	−25.414
	Mn_20_⋯N_3_	0.091	0.391	0.115	−0.132	0.871	−41.416
	Mn_20_⋯N_37_	0.115	0.465	0.144	−0.172	0.838	−53.966
	Mn_20_⋯O_19_	0.073	0.379	0.104	−0.112	0.922	−35.140
	Mn_20_⋯O_21_	0.092	0.468	0.130	−0.144	0.907	−45.181

**D**	H_54_⋯H_41_	0.012	0.040	0.009	−0.007	1.225	−2.196
	H_57_⋯C_11_	0.006	0.020	0.004	−0.003	1.336	−0.914
	H_63_⋯O_58_	0.016	0.059	0.013	−0.010	1.22	−3.138
	H_56_⋯O_19_	0.033	0.114	0.029	−0.029	0.985	−9.099
	H_59_⋯O_18_	0.034	0.100	0.027	−0.029	0.927	−9.099
	O_21_⋯H_60_	0.024	0.102	0.023	−0.020	1.141	−6.275
	C_24_⋯O_55_	0.007	0.028	0.005	−0.004	1.386	−1.255
	Co_20_⋯O_58_	0.037	0.143	0.039	−0.043	0.916	−13.491
	Co_20_⋯N_3_	0.100	0.468	0.142	−0.166	0.852	−52.083
	Co_20_⋯N_37_	0.121	0.545	0.172	−0.207	0.829	−64.947
	Co_20_⋯O_19_	0.077	0.406	0.116	−0.130	0.889	−40.788
	Co_20_⋯O_21_	0.094	0.471	0.138	−0.158	0.872	−49.573
	Co_20_⋯O_55_	0.037	0.153	0.041	−0.045	0.93	−14.119

**Table 4:** NPA charges on the ligand donor atoms and the central metal ions in the complexes investigated, computed at BP86-D3(BJ)/def2-TZVP(-f) level of theory in gas phase.

Atom/metal ion	**A**	**B**	**C**	**D**
**M**_20_	1.2323	0.9308	0.9438	1.0003
**N**_3_	−0.4590	−0.4023	−0.3932	−0.3837
**N**_37_	−0.4502	−0.3540	−0.3452	−0.3457
**O**_19_	−0.7521	−0.7049	−0.6761	−0.7007
**O**_21_	−0.7058	−0.6312	−0.6514	−0.6663
**O**_55_	−0.9494	−0.9434	−0.8375	−0.9144
**O**_58_	−0.9840	−0.9715	−0.8639	−0.9531

M = Cu(II), Ni(II), Mn(II), and Co(II).

**Table 5:** Global reactivity descriptors (GRD) of the complexes investigated in eV and eV^−1^ for global softness, calculated at M06/ma-def2-TZVP level of theory for the metal ions and M06/ma-def2-SVP level of theory for every other element, in the gas phase.

GRD	**A**	**B**	**C**	**D**
IP_v_	−0.13	0.29	0.24	0.28
EA_v_	0.08	0.06	0.02	0.02
*μ*	0.023	−0.17	−0.13	−0.15
*χ*	−0.023	0.17	0.13	0.15
*η*	−0.10	0.12	0.11	0.13
*S*	−9.63	8.61	8.82	7.63
*ω*	−3.31 × 10^−5^	1.73 × 10^−3^	9.42 × 10^−4^	1.41 × 10^−3^

## Data Availability

The data used to support the findings of this study are included within the article.

## Supplementary Materials

Supplementary Materials The Cartesian coordinates of atoms in the optimized geometries of the ligand (EDA2BB) in gas phase, along with those of its complexes in both gas phase and DMSO, all of which calculated at BP86-D3(BJ)/def2-TZVP(-f) level of theory, are presented in Tables S1–S9. The Harmonic vibrational frequencies of EDA2BB and its complexes in gas phase calculated at the same level of theory are presented in Tables S10–S14. The IR spectra based on unscaled vibrational frequencies of EDA2BB and its complexes in gas phase are shown in Figures S1–S5.
