# Supplementary material for: Structural Properties and Reactive Site Selectivity of Some Transition Metal Complexes of 2,2′(1E,1′E)-(ethane-1,2-diylbis(azan-1-yl-1-ylidene))bis(phenylmethan-1-yl-1-ylidene)dibenzoic Acid: DFT, Conceptual DFT, QTAIM, and MEP Studies
Source: Bioinorg Chem Appl. 2018 Sep 26;2018:4510648. doi: 10.1155/2018/4510648 (PMC6178161; doi:10.1155/2018/4510648)
Supplement: Supplementary Materials — The Cartesian coordinates of atoms in the optimized geometries of the ligand (EDA2BB) in gas phase, along with those of its complexes in both gas phase and DMSO, all of which calculated at BP86-D3(BJ)/def2-TZVP(-f) level of theory, are presented in Tables S1–S9. The Harmonic vibrational frequencies of EDA2BB and its complexes in gas phase calculated at the same level of theory are presented in Tables S10–S14. The IR spectra based on unscaled vibrational frequencies of EDA2BB and its complexes in gas phase are shown in Figures S1–S5. [file 4510648.f1.docx]

**Journal of Bioinorganic Chemistry and Applications**

**Supporting Information (SI) for**

**Structural properties and reactive site selectivity of some transition metal complexes of 2,2'(1E,1'E)-(ethane-1,2-diylbis(azan-1-yl-1-ylidene))bis(phenylmethan-1-yl-1-ylidene)dibenzoic acid: DFT, conceptual DFT, QTAIM and MEP studies**

**Fritzgerald Kogge Bine^1^, Nyiang Kennet Nkungli^1^, Taseh Stanley Numbonui^1^ and Julius Numbonui Ghogomu^1*^**

^1^*Laboratory of Noxious Chemistry and Environmental Engineering, Department of Chemistry, Faculty of Science, University of Dschang, P.O. Box 67, Dschang, Cameroon*

**Correspondence:* [*ghogsjuju@hotmail.com*](mailto:ghogsjuju@hotmail.com)

**A. Cartesian coordinates of atoms in the optimized geometries of** $\boldsymbol{EDA}\mathbf{2}\boldsymbol{BB}$ **ligand and complexes**

**SI Table S1** Cartesian coordinates of atoms in the optimized geometries of $\left[ Cu\left( EDA2BB \right)\left( OH_{2} \right)_{2} \right]$ complex in gas phase

65

C -2.83287153674721 2.83466181690238 -0.96108809884339

C -1.46146612064720 3.00395801347947 -0.30485616148253

N -0.71514633581593 1.72445334951166 -0.35272462798203

C 0.48796232213123 1.69894127785010 0.13266190860051

C 1.09006858114860 2.90711808101691 0.77546927591994

C 0.67774221542249 3.33713730236999 2.04440224011449

C 1.26695128301157 4.45968499485470 2.62757042090712

C 2.25745347828377 5.16739766384271 1.94096236329460

C 2.66909783932417 4.74102147566470 0.67563751113866

C 2.09768462913605 3.60635300243119 0.09766743554796

C 1.36755591052956 0.51374202130784 0.07270888234883

C 2.27567257658256 0.30336495610663 1.13110044728321

C 3.14223189556190 -0.78429237965514 1.12554040072107

C 3.14284891295246 -1.66345151237663 0.03722251444270

C 2.26702757413124 -1.45727288595121 -1.02727450527941

C 1.37524825314927 -0.37904095790245 -1.02183787303460

C 0.40841687170789 -0.19685811985223 -2.16323197911325

O 0.67169416886252 0.56565479800250 -3.10093492702215

O -0.68199445043328 -0.89257302875264 -2.01359413121109

Cu -2.02135132271394 0.24159691875931 -1.02278459582576

O -3.40864900482424 -0.65928769212637 -2.08348830910651

C -4.53110220566312 -1.17414044643396 -1.65441295509074

O -5.37032356614068 -1.67577420302234 -2.40430915192490

C -4.76145010096143 -1.17784385623601 -0.16014805876722

C -4.77355973761463 0.00061511064797 0.60712073079675

C -4.96988729741410 -0.07176247580192 1.99362241533659

C -5.15958548850484 -1.30434786173927 2.61696305510647

C -5.18150626042098 -2.47297293064635 1.85148806311445

C -4.99337338432604 -2.40487280223361 0.47348557127832

C -4.55386679536500 1.32925822533406 -0.03709603689455

C -5.70016307912917 2.24612194941885 -0.23377756621824

C -6.70551328399498 2.40182336655097 0.73627778732231

C -7.78705652024563 3.24773387179439 0.50045432220450

C -7.90282120670899 3.91926162355666 -0.71988527917122

C -6.93215291615822 3.73625062007261 -1.70790504844179

C -5.83500676165833 2.91213954061343 -1.46699166258124

N -3.35611173422314 1.57642332082509 -0.44856591085323

H -5.01353560286121 -3.30712302157481 -0.13780676690792

H -5.28725244668236 -1.35036640230084 3.69906170518740

H -5.33862106845833 -3.43928642915226 2.33212354046549

H -4.93786893847890 0.83938771855676 2.59307389584176

H 2.26896153363634 -2.13568709228584 -1.88120781481763

H 3.82498059557169 -0.94017569231121 1.96094332054935

H 3.83267270418096 -2.50817196266272 0.01607390335210

H 2.28871888063759 1.00403928849673 1.96608998800710

H -0.09233326687841 2.77975421604587 2.58078036087304

H 0.95276095613244 4.78177831805398 3.62105953943778

H 2.71256332559617 6.04806470120373 2.39534896150906

H 3.44562041190399 5.28749679325450 0.13936030108461

H 2.42566226381760 3.25623003232465 -0.88217526320614

H -7.03460901858226 4.22687198465467 -2.67625009854236

H -8.55312546551425 3.36981788274091 1.26688482888584

H -8.75907850444675 4.56801511918633 -0.90791343186586

H -6.64376310790567 1.85318337546913 1.67430000684516

O -1.40880924626933 -1.54463946818355 0.54071068436344

H -0.95697700633926 -1.74251321935063 -0.31728051768465

H -0.68244152071417 -1.24879696389173 1.11731648883425

O -2.03099294356105 1.38224482239806 -3.63018763334490

H -1.09254566974274 1.07304671093942 -3.66370844028597

H -2.55011397783564 0.55334102479651 -3.57478980478836

H -1.59556968565697 3.31380367469724 0.74082665532454

H -0.88492986722972 3.79074029115405 -0.81288217684172

H -2.70531823063471 2.72930331055948 -2.05191363705928

H -3.48139990515143 3.69291025336700 -0.73266053632741

H -5.10382564075774 2.73874197563057 -2.25585665552332

**SI Table S2** Cartesian coordinates of atoms in the optimized geometries of $\left[ Ni\left( EDA2BB \right)\left( OH_{2} \right)_{2} \right]$ complex in gas phase

65

C -2.87433567644401 2.69129639562518 -1.74832149629411

C -1.51788108157618 2.97910585917268 -1.10221541641572

N -0.85866108189533 1.67648543323126 -0.80030538916024

C 0.17699857951642 1.68167924693544 -0.00308065170344

C 0.56156609176695 2.92469985896247 0.73160013304015

C -0.18014132028570 3.34390184004968 1.84630378005589

C 0.19751773024321 4.48998535540932 2.54671031597061

C 1.30889613531544 5.22949062665360 2.13173663284197

C 2.05037775916796 4.81195123239434 1.02374795101744

C 1.68604579330252 3.65609151337160 0.33045869320450

C 1.05438224515842 0.52352351736903 0.21374467838191

C 1.69873333090366 0.37872959940605 1.45998139613088

C 2.57520132837123 -0.67509021301750 1.69258325162227

C 2.86476861181599 -1.57939844340826 0.66376610641899

C 2.26083896190697 -1.43399177223117 -0.58386193528605

C 1.34948968682802 -0.39900060297370 -0.81612320029014

C 0.63837073418754 -0.29851023401248 -2.13475283821264

O 1.13002844082421 0.27175881842201 -3.10778345422741

O -0.53282765209481 -0.89379937243192 -2.10008668562944

Ni -1.91555030758010 0.27996723879906 -1.56873504822959

O -3.06509731473395 -0.82101663780978 -2.52579510655851

C -4.17255809404482 -1.35241928707269 -2.07045936051064

O -4.97142984260306 -1.93778966110435 -2.79990690940027

C -4.45563758388196 -1.29642300280297 -0.58562776774382

C -4.64073404211042 -0.09785308193018 0.13639791591970

C -5.09631842428233 -0.15347461283885 1.46830135909796

C -5.28474961409440 -1.37518445192246 2.10426947168999

C -5.07032102056149 -2.56186059809461 1.39675188134861

C -4.69218673686356 -2.51663939244154 0.05770116736764

C -4.35859499034266 1.23523323041515 -0.42451303658910

C -5.28001966331742 2.34374225128442 -0.06248712682199

C -4.81923102326696 3.48614965243493 0.60988277566439

C -5.70986297771324 4.49961713579838 0.96503979974212

C -7.06434364533561 4.38440864751103 0.64210765584814

C -7.52955703559100 3.24542279396629 -0.02161786887246

C -6.64564197653857 2.22165721315898 -0.36040431246938

N -3.30530586244985 1.42727539054575 -1.15635250586035

H -4.58306947734481 -3.43455411594847 -0.51882935976285

H -5.60397545943654 -1.40257393237957 3.14645986295841

H -5.21933087593832 -3.52553519894816 1.88513434802608

H -5.27567465936433 0.77720777941849 2.00731403328618

H 2.48603691706077 -2.13170141785615 -1.39083831949089

H 3.04400972913832 -0.78589337589491 2.67043547609428

H 3.56685433656800 -2.39633128355865 0.83433685068668

H 1.49300603063250 1.09911686254594 2.25136029087034

H -1.04215572118518 2.75676559344287 2.16896840323060

H -0.37435851375505 4.80422585405753 3.42066752996474

H 1.60098713812602 6.12746485265358 2.67712817111975

H 2.92146911506631 5.38330119200079 0.70136519705902

H 2.27018801722508 3.31479656340380 -0.52532240565402

H -8.58694826867444 3.15045688899423 -0.27029808463598

H -5.34625484799141 5.37860419198420 1.49840149743064

H -7.75932844761231 5.17915543129107 0.91525468747781

H -3.76495104522351 3.56478297182962 0.87916560111093

O -1.14086106113332 -2.10696415037207 0.52424539308217

H -0.95606131027857 -1.87318611503584 -0.41315698832355

H -0.31228271498490 -1.86910277076747 0.97437539321295

O -1.43080870479521 1.17269130316960 -4.27190384569786

H -0.52275387758355 0.84379399172597 -4.07456010518614

H -1.97363776903308 0.36177595592136 -4.22143694597806

H -1.64349548727807 3.56553935709335 -0.18381395383390

H -0.86896921991849 3.54671476014467 -1.78380802285568

H -2.73735492298039 2.50923972821882 -2.82502450907084

H -3.58742558069217 3.50950023015130 -1.58509431554858

H -7.00355482031446 1.32322075588992 -0.86441086466005

**SI Table S3** Cartesian coordinates of atoms in the optimized geometries of $\left[ Mn\left( EDA2BB \right)\left( OH_{2} \right)_{2} \right]$ complex in gas phase

65

C -2.80704046309411 2.87011469372575 -0.97072774305395

C -1.44429844235552 2.89798288994249 -0.28532653253523

N -0.74318288034520 1.58547974072643 -0.45374580905924

C 0.49222312326007 1.54266931322524 0.04717741792167

C 1.00191640653662 2.71428212149058 0.82944852501189

C 0.55917671983704 2.96409451859418 2.13709164524986

C 1.05428931715238 4.05173526130562 2.85794339055291

C 1.99343480251257 4.90760486337591 2.27617969918785

C 2.44352996689270 4.66321988055278 0.97600293720248

C 1.95759961665094 3.56813962986147 0.25999006844519

C 1.47655958222600 0.46012094907747 -0.12352179075893

C 2.52696656340335 0.36773237486409 0.82361894255762

C 3.55764347173659 -0.55509534418067 0.69126927173310

C 3.59898114425232 -1.40613105218671 -0.41716737047670

C 2.58386447387975 -1.33536039839648 -1.37052041786316

C 1.52730689529852 -0.43393452715243 -1.22738132504264

C 0.40078172643207 -0.45138768101708 -2.20660261731185

O 0.45949237938183 0.12573377972563 -3.30625820280382

O -0.64105750342913 -1.08682847050502 -1.73712882197554

Mn -2.04546848306363 0.24721124637819 -1.16026070106269

O -3.42389545791982 -0.84798060411925 -1.98311768323555

C -4.55107741168941 -1.36873946391811 -1.53049828675949

O -5.32854456825855 -2.00114397961054 -2.23783096566350

C -4.81280432003200 -1.19355830910844 -0.05653581667029

C -4.78999089280670 0.06603238176644 0.56509793227475

C -4.95386043476171 0.14231350502517 1.95823084701639

C -5.15829382840055 -1.00828850747718 2.71923527949663

C -5.22614405209585 -2.25360583621200 2.08953045851263

C -5.05956380518656 -2.33963961154169 0.70944368372791

C -4.57131217587144 1.33951186165320 -0.18466468689607

C -5.68911604261877 2.27250242092064 -0.37742765458311

C -6.78453125932678 2.32305329618065 0.51180972601300

C -7.84991389960238 3.19046955902121 0.28556941160164

C -7.86889504803588 4.01274999385389 -0.84454748700939

C -6.81612058681130 3.93984623443089 -1.76189641586435

C -5.74518638883879 3.08085401662145 -1.53615767431334

N -3.35587611677389 1.54451228197449 -0.65435238997241

H -5.10734577290115 -3.30176813797571 0.19877897769575

H -5.26841308279912 -0.93002089009524 3.80168655080722

H -5.40088527239794 -3.15665022877761 2.67579880537659

H -4.89855896822124 1.11527319978704 2.44952007636455

H 2.59977906405830 -1.98959928011777 -2.24309026535656

H 4.33859422795135 -0.60189237127641 1.45091353938020

H 4.41583382982187 -2.11799110557411 -0.53953024574460

H 2.52522628163834 1.04115435461334 1.67940350339747

H -0.16795708814137 2.29121174929967 2.59643568013631

H 0.70954064354395 4.22898992579757 3.87752037092657

H 2.37839102815986 5.75942086845623 2.83784204429905

H 3.18120304020769 5.32414225498762 0.51918436360449

H 2.31865265271486 3.36357103017953 -0.74903569837957

H -6.84027761295577 4.54070335508618 -2.67199920241270

H -8.67967767058583 3.21566812641875 0.99319062654214

H -8.70916488637037 4.68436253329770 -1.02329420783942

H -6.80441807362146 1.66803408508747 1.38056845465963

O -1.74443448844139 -0.95722359123126 0.66004753102538

H -1.06303862853491 -1.47228805512655 0.15671285668043

H -1.22446026986178 -0.38675790633656 1.25793282541777

O -2.04292880787235 0.96189239032018 -3.17552531212685

H -1.12534084851961 0.63598780907088 -3.46656994023012

H -2.67996185129097 0.26949473183844 -3.47160762695841

H -1.59341632313307 3.09406860673297 0.78514691854293

H -0.81828176209904 3.70880778281400 -0.68421027315218

H -2.69713236497921 2.97794731903870 -2.05833303778150

H -3.44180456411268 3.67851996090954 -0.58362886553229

H -4.96578759939178 2.99090184390722 -2.29105742293653

**SI Table S4** Cartesian coordinates of atoms in the optimized geometries of $\left[ Co\left( EDA2BB \right)\left( OH_{2} \right)_{2} \right]$ complex in gas phase

65

C -2.85581142953955 2.88432616412154 -1.09187653369956

C -1.47839954145209 3.00266364828533 -0.44640273460542

N -0.78973689818642 1.67667956927208 -0.47681704222697

C 0.40119419664815 1.63616338270876 0.07371031456830

C 0.96552073964899 2.84633007777004 0.74865857487689

C 0.52362622074531 3.24382130854499 2.01890617102973

C 1.08510754922125 4.35986441128733 2.64076547351879

C 2.08369242187192 5.09342521842054 1.99417454506090

C 2.52861517044028 4.69952826263727 0.72966834715578

C 1.98024478038334 3.57394664709875 0.11255392875678

C 1.29993119871591 0.46762831005990 0.06531312582493

C 2.18886506584269 0.29608023139049 1.14976118764786

C 3.09957992792383 -0.75385655467109 1.17590369071460

C 3.17267080375325 -1.63940259961955 0.09526580225284

C 2.32036520690885 -1.47536012057363 -0.99514835590975

C 1.38309715456235 -0.43809143966588 -1.01814941253358

C 0.42571092403448 -0.30438393837189 -2.16371413318394

O 0.69776724190180 0.36495357441548 -3.16892372016402

O -0.69509670355522 -0.93611382643088 -1.94320156319027

Co -2.00222870908068 0.35789611900235 -1.20854728025056

O -3.31235005580466 -0.69223131542899 -2.13511058503777

C -4.43406748866667 -1.23106852737018 -1.72071543869683

O -5.23782323533233 -1.77986258336563 -2.47358614695869

C -4.67866601056197 -1.20652926695560 -0.23224187294490

C -4.67485847049652 -0.01335628740321 0.50818104109889

C -4.83034058925727 -0.06608548746638 1.90231293763581

C -5.00857029894728 -1.28669443298192 2.55131949039772

C -5.06494781324327 -2.46728680939847 1.80631313380839

C -4.90641227877720 -2.42174102899586 0.42372532476482

C -4.48893284376356 1.31022587857644 -0.14997985435261

C -5.62366590037009 2.25882687985456 -0.22760866933195

C -6.53841678053690 2.44450047611544 0.82440604128320

C -7.61672228997791 3.31549931490044 0.68070016290341

C -7.82330685960330 3.99297562668140 -0.52374866319655

C -6.94642972588184 3.78471007638480 -1.59141162472140

C -5.85791804433049 2.92833930972214 -1.44593380539291

N -3.33209310549207 1.55798800292576 -0.69453162321788

H -4.94377527324069 -3.33237793914821 -0.17436635409681

H -5.10462019007643 -1.31337598469468 3.63742754185042

H -5.22106121131974 -3.42405947616885 2.30622475844689

H -4.76993752591970 0.85137512889900 2.48913518252204

H 2.37686006764165 -2.15492501579297 -1.84621209846102

H 3.76288450524820 -0.87461261670204 2.03265556259846

H 3.89891222062684 -2.45308628934055 0.09988411185040

H 2.15451366969634 1.00119865221837 1.98000195575456

H -0.25269207224543 2.66639192850585 2.52476906330799

H 0.74379190692142 4.65597555356817 3.63344505756939

H 2.51885994964848 5.96797958219368 2.47892367855441

H 3.31215327733930 5.26525515664464 0.22429753188407

H 2.33531665799175 3.25007230485485 -0.86692568545123

H -7.11932438327055 4.27794736633266 -2.54853115561350

H -8.30876633319806 3.45510183471014 1.51197882471865

H -8.67591921086620 4.66346688984796 -0.63673703896329

H -6.41246537276275 1.90004449691825 1.75755959651234

O -1.63778958083387 -1.17049146271788 0.53321111756259

H -1.10135869003948 -1.54304239341442 -0.21235854858136

H -0.96984689649126 -0.79982839460356 1.13744573083336

O -1.92924026286877 1.21108819200798 -3.40824782661564

H -1.00364074800866 0.89692045270896 -3.59767094888404

H -2.50124250172039 0.43263120797873 -3.58950597847847

H -1.59231177250486 3.32677114407364 0.59617949475410

H -0.86045204673963 3.75267464002071 -0.96006004997708

H -2.77384337118705 2.91711180560097 -2.18550804296108

H -3.51743550145450 3.68750969612354 -0.73959846226503

H -5.20523588011100 2.73420865789834 -2.29663738205511

**SI Table S5** Cartesian coordinates of atoms in the optimized geometries of $\boldsymbol{EDA}\mathbf{2}\boldsymbol{BB}$ ligand in gas phase

60

C -2.88126114408653 2.09169538823951 -0.44862233571713

C -1.66015069810273 2.14171598666280 0.47575982627526

N -0.65557689097747 1.17235226286731 0.06802237156390

N -3.78970863986718 1.02429162457295 -0.05948670447330

C -5.04363282057311 1.09952298831712 -0.32664804606823

C -5.68889105687658 2.26547378822442 -1.01209903218978

C -5.91589905984345 -0.03514259272553 0.06016615829026

C -6.01625577642632 3.42636591687171 -0.29760045284706

C -6.59658538691414 4.51549445194749 -0.95113417681656

C -6.85181153430440 4.45440726831853 -2.32340366637596

C -6.52829177726369 3.29883513400797 -3.03953136085232

C -5.95271349090598 2.20716162475594 -2.38680010811648

C -7.30166437832479 0.14992864020848 0.18995669770985

C -8.12618126015788 -0.88189755062634 0.63663928797174

C -7.57362151730041 -2.12103293628731 0.96408384336954

C -6.19841448539855 -2.32483555626409 0.83005331712951

C -5.37048685907783 -1.30104912635527 0.36533611676084

C -3.91293486387242 -1.60615059740651 0.19199510312749

O -3.20745497870061 -1.88456924770633 1.17490184724904

O -3.52891094947443 -1.67905127595716 -1.06767747204361

H -2.49820965559464 -1.78614486001013 -1.11603806861010

C 0.58662935746831 1.36111839178980 0.33368582322797

C 1.11952626544616 2.57031074097934 1.04046781197026

C 1.37677191131447 2.51607699650875 2.41661947803048

C 1.84646136590479 3.64558317794249 3.08938375357742

C 2.06964636223868 4.83592634809660 2.39209902705106

C 1.82043873174785 4.89365141680294 1.01862777192886

C 1.34615840563664 3.76609102164380 0.34486825033732

C 1.56110356903972 0.32241130175659 -0.07748377293658

C 1.13573398161894 -0.98066760421987 -0.41543990161797

C 2.05583515635360 -1.91192796999614 -0.90077100601077

C 3.40663725637897 -1.57862206017687 -1.02413233562226

C 2.92396868524391 0.63789860022375 -0.19680093807030

C 3.84147739772160 -0.30213072192124 -0.66469355040145

C -0.28725388727978 -1.42468357030439 -0.25525481783526

O -0.96441505550709 -1.73787541746382 -1.24757061445544

O -0.66103222217075 -1.57248440104067 1.00093985575047

H -1.67628207162693 -1.77990053414875 1.04438610999191

H -5.75777467614816 -3.29171022907055 1.07567359373955

H -8.21040110680557 -2.93254675766045 1.31813663866566

H -9.19940651697894 -0.71513918630240 0.73535120955404

H -7.72858756524857 1.12339045465997 -0.05168337455019

H -6.85109817015521 5.41354552379269 -0.38650327854001

H -5.81867190316638 3.46926781936952 0.77497772371653

H -7.30447574394816 5.30541804554882 -2.83375688581478

H -6.72766034871979 3.24562761214439 -4.11071223353396

H -5.70421651679551 1.30078007853769 -2.94123498834425

H 1.70710113581826 -2.90918002746961 -1.17137828032933

H 4.11651128683983 -2.31896178367308 -1.39478692075678

H 4.89490734996852 -0.03460519135654 -0.75448615341182

H 3.25875740662056 1.64038586180483 0.07002443829917

H 1.20652444893681 1.58313812612916 2.95635267746132

H 2.04165714431563 3.59505584342956 4.16146481976496

H 2.43955118717176 5.71684963266615 2.91814967466504

H 1.99684771097983 5.81919286662216 0.46877729868785

H 1.15380447086238 3.80663748992547 -0.72874999148400

H -3.37406606822278 3.07875416964845 -0.46591644094516

H -2.53282926880425 1.88674726963570 -1.47562006400657

H -1.26259611776026 3.17045788152737 0.51054336543240

H -1.98727612424599 1.88682745196287 1.49857308147768

**SI Table S6** Cartesian coordinates of atoms in the optimized geometries of $\left[ Cu\left( EDA2BB \right)\left( OH_{2} \right)_{2} \right]$ complex in DMSO

65

C -2.81467113908041 2.82797509271255 -1.06105520653636

C -1.46693265167478 3.02257000678400 -0.37132027797933

N -0.70800405499233 1.75060566607455 -0.39924032442480

C 0.49172659293161 1.72208045893000 0.09476180950025

C 1.09947830245555 2.91764719294262 0.74821010521854

C 0.60816993625993 3.40075362289403 1.96996342844265

C 1.21208244845666 4.50399861627714 2.57508152286789

C 2.29280501080101 5.14033326637493 1.95555621529158

C 2.78318275389271 4.65965961793621 0.73727592266095

C 2.19949297813236 3.54055312289422 0.14072433337481

C 1.34667690632009 0.51945267641807 0.03889156675180

C 2.21581770132782 0.27196401754303 1.12121125791268

C 3.06178929405252 -0.83277207062667 1.12126723209039

C 3.08315615187069 -1.68858020809572 0.01465712726106

C 2.24614336352192 -1.44396428058868 -1.07460625389890

C 1.37029573192702 -0.35191086031909 -1.07120856142903

C 0.45963547806709 -0.15440681845907 -2.25434905600697

O 0.82465332011574 0.50045450799551 -3.24835742766533

O -0.68547417234493 -0.75134359480581 -2.14770250218462

Cu -1.96164383706047 0.22023794937592 -0.99603221682754

O -3.35429907181867 -0.67672444332135 -2.15479100091626

C -4.50181100195937 -1.14152461335302 -1.75829994070418

O -5.36634594662566 -1.58419989366693 -2.53348682507534

C -4.72893018172441 -1.18649496348092 -0.26558354624083

C -4.70027358945921 -0.02618945310479 0.53056043269717

C -4.80281233782740 -0.13568474244021 1.92541810626773

C -4.94628468619854 -1.38933523649482 2.52563607680406

C -5.02170946857430 -2.53794927171348 1.73209043633880

C -4.92116773818159 -2.43299664615594 0.34479342764966

C -4.51568574614359 1.31776960447788 -0.09854833300073

C -5.67394413436797 2.22651152244461 -0.22461597984953

C -6.63934646948653 2.32775768005827 0.79400479697800

C -7.73813611866500 3.17090255480726 0.63870336489220

C -7.90935464330105 3.89383134911953 -0.54671446492366

C -6.97586603329989 3.76892102617668 -1.58040272561874

C -5.86145914125285 2.94745363846160 -1.42031555914564

N -3.33300307878287 1.56492195746186 -0.54890381241424

H -4.96069546963029 -3.32650365491239 -0.27920278260890

H -4.99931780909737 -1.46382708759962 3.61193135750996

H -5.14411700827867 -3.51728939995797 2.19553282695829

H -4.74013144723250 0.75812709653823 2.54650965576116

H 2.27047964564396 -2.10569456918331 -1.94153093521931

H 3.71156750672475 -1.01956321716915 1.97630824565752

H 3.75758537111075 -2.54554462100212 -0.00351178448647

H 2.21079249893582 0.95209711053382 1.97290382568666

H -0.22891196727356 2.90045705261846 2.45906456412809

H 0.83778110339016 4.86617671456962 3.53321261583931

H 2.75736792210098 6.00750924842732 2.42650707789557

H 3.62937031642995 5.14978444418674 0.25446669309429

H 2.59102221506292 3.15049970560632 -0.79990243809050

H -7.11785306387185 4.30759737572463 -2.51769127368107

H -8.47005160555620 3.25534475099605 1.44250672497022

H -8.77711691469256 4.54286387870673 -0.67016280473622

H -6.52899959332943 1.75538560980591 1.71324947905927

O -1.67495136537618 -1.14637663559452 0.81636677829098

H -0.82244703924261 -0.99808660843869 1.26625553596845

H -2.34931758235466 -0.98157439570963 1.50437472571885

O -2.01005655542149 1.11675502555621 -4.11251831784929

H -1.07724029095780 0.88134568384359 -3.89639634861320

H -2.52530152071301 0.43059677765402 -3.63478893785045

H -1.64374193945941 3.32547174388194 0.66831926299614

H -0.89008689952141 3.81859323492585 -0.86235465136842

H -2.67578445824042 2.72776349304798 -2.14990992292815

H -3.47792868445273 3.67798957220625 -0.85413539737944

H -5.15833913200796 2.83005700920376 -2.24390705288152

**SI Table S7** Cartesian coordinates of atoms in the optimized geometries of $\left[ Ni\left( EDA2BB \right)\left( OH_{2} \right)_{2} \right]$ complex in DMSO

65

C -2.88017801891554 2.85276772957849 -1.63997549817225

C -1.54005078030048 3.11680513376142 -0.95926541111268

N -0.86393160271180 1.80183566276850 -0.72648778551056

C 0.19289769550314 1.78423804490531 0.04182576594010

C 0.60514180358835 3.02842047413020 0.75937547450928

C -0.11262847581886 3.46408979412332 1.88369185204399

C 0.29368810779640 4.61141759540603 2.56584131536530

C 1.40579073208533 5.33442229620140 2.12103624672085

C 2.12110882042667 4.89938905403201 1.00141491568084

C 1.73047357734079 3.74077308541471 0.32591940614326

C 1.06823398805053 0.62015373390225 0.25917492343608

C 1.72609237208120 0.52500411556975 1.50385634963253

C 2.64128673397725 -0.48932580548278 1.76263372056069

C 2.97241438595271 -1.39991504071780 0.75446069683764

C 2.36319366292646 -1.30104694552961 -0.49569807881134

C 1.39702448723212 -0.31893527329188 -0.74576754727238

C 0.71595486495321 -0.36063166737641 -2.07730180590163

O 1.31319635788083 -0.16155708392473 -3.14044570887580

O -0.53845667801658 -0.75181367821766 -2.02306816861645

Ni -1.90132559954092 0.42896787457331 -1.51910639774364

O -3.02100318938460 -0.64627496844897 -2.57412243586151

C -4.14043585194115 -1.20118897220533 -2.19527660662926

O -4.90173275258666 -1.76130390764656 -2.99470432224834

C -4.46400173994068 -1.22345733160213 -0.72145434369937

C -4.61655060990460 -0.06704396016614 0.07548533623246

C -5.02829866885579 -0.20485250880366 1.41363186686030

C -5.21422906118823 -1.46329751298527 1.98038500786789

C -5.04745762321668 -2.60757699717235 1.19575905351349

C -4.70663593141509 -2.48246336580191 -0.15245126649529

C -4.36265917647990 1.30810662998918 -0.40971760306103

C -5.30583277982784 2.36828845131988 0.01588289071318

C -4.85777751874036 3.50125323654718 0.71429360308137

C -5.77048338554940 4.46772724345509 1.13681630822765

C -7.13144257893102 4.31559994423049 0.85374444855489

C -7.58264432831530 3.18464448828560 0.16447409508988

C -6.67705669936274 2.20432003352858 -0.23985076715936

N -3.30805631606198 1.55433777729288 -1.12205708106989

H -4.62849940181601 -3.36831525098207 -0.78323061804751

H -5.49815867646891 -1.54934969450652 3.02912649060118

H -5.20222526155861 -3.59752091187622 1.62551288148982

H -5.17714772685929 0.68974381738402 2.01811047071702

H 2.62479959772613 -1.99962926833332 -1.29109948711598

H 3.11270106313310 -0.55627027021544 2.74313639893652

H 3.71137629604552 -2.18060200503411 0.93746894831143

H 1.50501314249543 1.25843189621104 2.27805091601856

H -0.97472378777344 2.89345864902356 2.23359550662644

H -0.25867888257786 4.93974110636657 3.44688298273601

H 1.71824354447186 6.23448265623326 2.65176912455634

H 2.99142693779871 5.45809548382199 0.65550611005392

H 2.29524926552486 3.39009123885962 -0.53888182200677

H -8.64434898861165 3.06151902293108 -0.05116656705045

H -5.41787633865769 5.33703475557735 1.69232748603518

H -7.84327353484938 5.07466445534721 1.18007663934419

H -3.80123138576175 3.61011314764816 0.96087977299701

O -1.32944068527136 -2.27549242621792 0.34921162026578

H -0.99767023287349 -1.78788968431340 -0.43983130371143

H -2.29887663367837 -2.26877011633300 0.23522968475772

O -1.15167035773150 -0.21585445659304 -4.90926591984748

H -0.33021634619889 -0.23117361262352 -4.36746970483272

H -1.84204135017424 -0.44736616986390 -4.25243182617603

H -1.69881064674274 3.63070652004608 -0.00515981380359

H -0.89080759297872 3.74796227674320 -1.57970806267058

H -2.75652805987099 2.74299242167410 -2.72646086724430

H -3.60172586364601 3.65458251946243 -1.44512551748009

H -7.02695935588351 1.31496990992048 -0.76523610223109

**SI Table S8** Cartesian coordinates of atoms in the optimized geometries of $\left[ Mn\left( EDA2BB \right)\left( OH_{2} \right)_{2} \right]$ complex in DMSO

65

C -2.79705518379224 2.86758473058186 -1.02795456503621

C -1.42225636581455 2.92451552513704 -0.36657973463762

N -0.71380132218839 1.61152624889655 -0.50539910565111

C 0.50174999818427 1.56010790869927 0.01679250905929

C 1.02115305002945 2.73074878560756 0.79115375508121

C 0.56369341892644 2.99120442799170 2.09159475675844

C 1.06926506454111 4.07594127278400 2.81066170963254

C 2.02830663695967 4.91442847046937 2.23272828472413

C 2.48915137096917 4.65711190246930 0.93763684362704

C 1.99482098453852 3.56397441044286 0.22238774035643

C 1.45312803795050 0.44262322174093 -0.11751155516517

C 2.45238156777044 0.30676198884133 0.87662508781262

C 3.45717258659728 -0.64941990023109 0.78168252878665

C 3.52121107074654 -1.48716054585222 -0.33730210212933

C 2.55483334517528 -1.37191525058650 -1.33659238293788

C 1.52045336974951 -0.43578368458837 -1.23100131711144

C 0.45110317172387 -0.42710859614027 -2.27417806926083

O 0.59283407212082 0.16073029614230 -3.36993136495747

O -0.61521065563774 -1.07384177184455 -1.91130509628551

Mn -1.98779474534900 0.25415159074409 -1.18152997370801

O -3.41917399125860 -0.88230080229470 -2.03908180182372

C -4.55122606081521 -1.34658138673606 -1.59295864029285

O -5.37640273246161 -1.93503096526864 -2.31321894887892

C -4.79987172127613 -1.20757689731357 -0.11404745471738

C -4.73205839746656 0.04073892609938 0.53149767793508

C -4.81562102960992 0.08838321927513 1.93517598882036

C -4.99383678113489 -1.08060530359254 2.68106998308488

C -5.11413397321616 -2.31073209089962 2.02892256100512

C -5.01957533440895 -2.36939874093839 0.63813888117672

C -4.53176519547303 1.32342419182594 -0.21238379794003

C -5.66154505631177 2.24819257272663 -0.36540157758508

C -6.72198370552063 2.28443823323332 0.56693949422726

C -7.80857089305187 3.13690878644550 0.38067109901447

C -7.87947439497448 3.95918198432105 -0.74900239572601

C -6.85586572759134 3.90696149603491 -1.70229777565889

C -5.76440949390247 3.06240105027668 -1.51713498114537

N -3.32577968521058 1.53072950350709 -0.70155336329289

H -5.08463208285418 -3.32771435376199 0.12154531102937

H -5.03792528102394 -1.02494879352309 3.76904777814491

H -5.26260695936396 -3.22511658039344 2.60414135623947

H -4.72420293202525 1.04801961547293 2.44563669099097

H 2.59288007663377 -2.02164913160185 -2.21235690376611

H 4.19912852200184 -0.73336757532358 1.57635911183804

H 4.31870997353513 -2.22490912715199 -0.43222842365829

H 2.42825929083617 0.96899969614153 1.74089256349693

H -0.17984923337217 2.33350672146592 2.54575488460716

H 0.71519333634139 4.26508140853503 3.82490741065616

H 2.41968504284689 5.76432121059629 2.79339572399612

H 3.24009128467847 5.30551572016970 0.48419737705506

H 2.36188303077959 3.35363388416098 -0.78344772498285

H -6.91559614897181 4.51697168594321 -2.60469425864162

H -8.60893959116002 3.15503594015766 1.12166503697350

H -8.73353025901876 4.62155452768313 -0.89503304363087

H -6.69606626049844 1.64279609071329 1.44589630051960

O -1.86086178435305 -0.76551941121947 0.65376253282260

H -1.02658590172176 -0.55786696649418 1.12038535367577

H -2.58734995635837 -0.51753455733625 1.26594507236321

O -1.96252869561730 1.01017127981528 -3.21314795057308

H -1.06265173548847 0.68280153699475 -3.52686734814681

H -2.61388391954184 0.38130917967433 -3.59224909818327

H -1.55506945387605 3.14430704871552 0.70003224227926

H -0.81071376732938 3.72671097092805 -0.80137853625085

H -2.71523655767290 2.98239926179920 -2.11575563211170

H -3.43594674811099 3.66281476129851 -0.62501300953387

H -5.00397162881141 2.99959453853334 -2.29393584436929

**SI Table S9** Cartesian coordinates of atoms in the optimized geometries of $\left[ Co\left( EDA2BB \right)\left( OH_{2} \right)_{2} \right]$ complex in DMSO

65

C -2.90603636531870 2.80375144921903 -1.44848831423974

C -1.57196728571892 3.16032766951202 -0.79769479233944

N -0.80699576301236 1.89492920818289 -0.63368575732145

C 0.30437990602911 1.90003684897918 0.04230176476969

C 0.84415498373910 3.15078451167348 0.64915904212036

C 0.23739619050832 3.73662406482942 1.77040468748926

C 0.78193158307844 4.89141108735281 2.33486372984080

C 1.92601058346962 5.47228718746616 1.77817204518119

C 2.53473794635333 4.88733922208228 0.66346810816900

C 2.00367735025605 3.72222622371898 0.10602440102574

C 1.06980951880257 0.66253086328333 0.31019821854787

C 1.55016415332501 0.48889111122909 1.62380918434305

C 2.21384324901094 -0.67477111491323 2.00080244572899

C 2.45791338860637 -1.67291007382691 1.05231220031394

C 2.05224290940867 -1.48444859852802 -0.26860088487591

C 1.35040349311284 -0.33183727882382 -0.65299819483943

C 1.01088583330671 -0.20040275791972 -2.11593212261433

O 1.92808960408397 -0.21576804934382 -2.95275676653640

O -0.24644836458506 -0.15374361994416 -2.44160251668569

Co -1.75549022453003 0.45195042116315 -1.42710944828844

O -2.82065167488719 -0.70266568835516 -2.53666473950633

C -4.00052041328582 -1.17840849420153 -2.26380683639684

O -4.78172364723897 -1.59023533639498 -3.13593077634180

C -4.39755656247153 -1.31966149380941 -0.81104860264238

C -4.63279191655234 -0.22624205721133 0.05189260516411

C -5.12396568189132 -0.46632971851144 1.34793051630841

C -5.30744182360570 -1.76524587040041 1.81533912461104

C -5.04068995537372 -2.84851160401374 0.97257143741426

C -4.61527896206117 -2.62247304677043 -0.33776811719215

C -4.36880474042544 1.17698073805751 -0.33391171814306

C -5.36759940183860 2.19414616869265 0.07094068920861

C -4.99634617055144 3.30889066139094 0.84074915816767

C -5.95860425932759 4.23674383866891 1.24029649114951

C -7.29534703201155 4.06398599704405 0.86706945756810

C -7.67190826416861 2.95082595998381 0.10755812125022

C -6.71595753156837 2.01064358831603 -0.27695099855271

N -3.26270724741934 1.46781026209749 -0.94910788207691

H -4.45374583033766 -3.46338923938986 -1.01303901193350

H -5.65828307189303 -1.93182475235326 2.83389856320934

H -5.18299368940259 -3.86982419067015 1.32710563664465

H -5.33269091376018 0.38164101611661 2.00046523914852

H 2.28419640617281 -2.23445687515137 -1.02540011429713

H 2.54539173771388 -0.79953434284039 3.03180432183935

H 2.98256682412172 -2.58610682407775 1.33477287131194

H 1.37300526662350 1.27190292773969 2.36077293689433

H -0.64746662373662 3.27745128222206 2.21421625636095

H 0.31225166921782 5.33605592943889 3.21297187549262

H 2.34680978419543 6.37748468826141 2.21766176864081

H 3.43047402172406 5.33382742506478 0.23013008399301

H 2.48474825052738 3.25543769625116 -0.75487033172558

H -8.71453649117228 2.81073498584006 -0.17971159820307

H -5.66441762574021 5.09211617891342 1.84928618097835

H -8.04604220175444 4.79205894782734 1.17683105362083

H -3.95952994218069 3.43054975319283 1.15607487671077

O -1.59053258916592 -0.87701315187056 0.32153366621765

H -0.73012835701243 -1.33693740933302 0.25381075675186

H -2.25791425185721 -1.58566117664567 0.21866238077139

O -1.60802003090735 0.31737745156873 -4.88391736210799

H -0.84521669303962 0.22967570264998 -4.26821347141365

H -2.31618402269171 -0.07131198697082 -4.32216846464032

H -1.74074986232853 3.62397596140405 0.18151389912000

H -1.00184503361349 3.86918090361808 -1.41299935910367

H -2.78854409779509 2.71064081068198 -2.53958343199392

H -3.67129637016920 3.56311029249945 -1.24985765512012

H -7.00858670698579 1.13560910603736 -0.85878665694627

**B. Harmonic vibrational frequencies for** $\boldsymbol{EDA}\mathbf{2}\boldsymbol{BB}$ **ligand and complexes**

**SI Table S10** IR frequencies for $\left[ Cu\left( EDA2BB \right)\left( OH_{2} \right)_{2} \right]$ complex in gas phase

The first frequency considered to be a vibration is 10

The total number of vibrations considered is 185

Mode freq (cm**-1) T**2 TX TY TZ

-------------------------------------------------------------------

10: 39.69 0.496215 ( -0.678850 0.164208 -0.091728)

11: 42.60 1.817181 ( 0.033368 -1.290338 -0.388709)

12: 51.55 2.511423 ( -0.644886 1.297706 -0.641486)

13: 56.18 1.334232 ( -1.147221 -0.030853 0.131009)

14: 63.58 0.090275 ( -0.200849 0.128914 0.182526)

15: 64.89 0.165107 ( 0.219629 -0.099073 0.327191)

16: 74.14 3.307428 ( 0.367539 -0.807715 1.587432)

17: 81.63 3.525010 ( 0.496692 -0.716863 1.662653)

18: 90.67 12.094772 ( -0.012789 -1.284266 3.231914)

19: 94.11 0.245941 ( -0.388123 -0.204370 0.231375)

20: 98.77 3.663339 ( 0.720257 -1.707974 -0.476857)

21: 107.25 4.900778 ( -0.447752 1.998717 -0.839897)

22: 113.10 7.147530 ( 0.375373 0.750768 -2.538301)

23: 118.86 1.676955 ( -0.403496 0.931615 0.803890)

24: 130.69 0.881772 ( 0.732217 0.014693 -0.587720)

25: 141.61 1.504245 ( 0.626897 -0.203435 -1.034340)

26: 148.38 1.004474 ( 0.101335 -0.115032 -0.990441)

27: 154.43 0.640457 ( -0.406931 -0.545970 0.420452)

28: 164.43 0.630130 ( 0.411370 0.219415 -0.642466)

29: 169.65 1.848021 ( 0.984865 0.015062 0.936928)

30: 188.23 1.243125 ( 0.399015 -0.891409 0.537868)

31: 199.18 2.637805 ( -0.231917 1.607375 -0.019056)

32: 210.56 0.631596 ( 0.541583 -0.380971 -0.439483)

33: 219.76 7.982228 ( 1.062392 -1.319571 2.261036)

34: 239.49 0.383193 ( 0.305854 -0.322044 -0.431200)

35: 242.77 10.820966 ( -3.022034 -0.605476 1.149641)

36: 254.05 0.657507 ( -0.232552 0.717388 -0.297962)

37: 269.96 6.775970 ( 1.676574 -0.208254 1.980328)

38: 284.24 4.994647 ( 1.908855 1.016758 0.563135)

39: 287.43 10.591668 ( -2.704929 -0.435233 -1.756587)

40: 297.52 27.902075 ( 2.380862 2.661635 -3.892206)

41: 320.72 6.050870 ( 0.760056 -0.108191 -2.336981)

42: 323.13 30.431055 ( -4.104185 2.897505 -2.278417)

43: 331.55 11.255150 ( 2.869536 0.434703 -1.682839)

44: 345.31 8.151099 ( 0.457525 2.307120 -1.618322)

45: 356.21 6.663306 ( 0.922560 0.887373 -2.241597)

46: 370.66 31.221530 ( 4.441090 -3.388311 -0.132668)

47: 372.92 3.150326 ( 0.513155 0.819605 -1.488371)

48: 383.07 58.410501 ( 1.282958 7.519559 0.469837)

49: 391.42 0.619549 ( -0.641669 -0.332703 -0.311638)

50: 394.28 2.345421 ( -1.053454 -0.979303 -0.525947)

51: 409.73 14.696412 ( 1.118645 -0.451857 -3.638801)

52: 416.53 0.922597 ( -0.439754 0.482100 0.704835)

53: 443.86 2.698901 ( -1.578941 -0.143830 0.430302)

54: 457.30 3.804191 ( 1.544243 0.314926 1.149055)

55: 463.03 3.473600 ( -0.864790 1.223259 -1.108773)

56: 478.90 34.933823 ( 4.943078 2.020862 -2.532967)

57: 484.05 23.445208 ( -4.742822 0.021760 0.974873)

58: 522.60 13.234445 ( -3.343527 -0.344776 -1.391547)

59: 560.33 4.427668 ( 0.739693 -0.553189 1.890636)

60: 562.25 42.300875 ( 1.182445 0.265048 -6.390027)

61: 564.65 74.505302 ( -0.190585 0.296281 8.624453)

62: 589.40 3.118166 ( -1.562949 0.622202 -0.536861)

63: 612.46 0.553494 ( -0.571608 -0.434758 0.194276)

64: 613.67 0.579019 ( -0.144503 0.011031 -0.747005)

65: 615.21 4.730264 ( -0.488792 -1.783453 -1.144833)

66: 637.60 14.898377 ( 2.499358 -2.372972 1.737984)

67: 642.54 6.423997 ( 1.407404 2.102217 -0.154574)

68: 656.27 34.360064 ( -2.334593 -2.663855 -4.670505)

69: 662.14 18.751409 ( 3.901145 -1.486561 -1.150051)

70: 677.10 197.709201 ( -0.710144 -10.454140 9.376346)

71: 680.90 16.284144 ( -0.705093 2.058356 3.398552)

72: 685.99 35.534841 ( -3.384542 -3.701092 3.222054)

73: 691.15 48.391694 ( -5.969444 -2.733415 -2.299104)

74: 694.66 44.655800 ( 3.935892 -4.415450 3.109397)

75: 704.14 105.437199 ( 2.962717 0.961196 9.784458)

76: 718.91 8.437412 ( 2.620466 -0.951048 0.816135)

77: 724.98 0.177856 ( -0.368958 -0.174889 -0.105546)

78: 729.06 6.809533 ( -0.360476 1.909806 -1.741330)

79: 733.03 35.717520 ( -4.230764 4.147636 -0.784394)

80: 749.39 45.723127 ( 3.861245 4.029183 -3.818325)

81: 763.67 34.826190 ( 5.147097 2.115541 1.964198)

82: 765.31 3.809529 ( 0.080325 0.741229 -1.803789)

83: 768.12 3.934329 ( 1.835530 -0.504356 0.557480)

84: 775.22 2.067608 ( -1.143076 0.818477 0.301797)

85: 780.96 23.290328 ( 2.167553 -2.268067 -3.667140)

86: 798.71 28.144146 ( 3.890812 2.627024 -2.470722)

87: 814.83 1.691328 ( -0.525784 0.904554 -0.772438)

88: 822.94 3.872047 ( -1.691356 0.997688 0.126405)

89: 829.95 0.204617 ( 0.138419 0.430625 0.004378)

90: 848.06 2.194440 ( 0.143048 1.234050 0.806906)

91: 859.24 0.815670 ( -0.864796 -0.005733 -0.260315)

92: 865.65 2.936001 ( 1.325695 0.153074 -1.074757)

93: 882.16 0.624260 ( 0.628827 -0.208205 0.430682)

94: 898.66 0.324492 ( 0.189948 -0.515893 0.149216)

95: 904.28 2.343670 ( 0.588193 -1.406930 0.135085)

96: 909.44 1.112046 ( 0.967232 -0.367386 0.203801)

97: 918.26 1.796377 ( -0.360550 -1.283540 -0.137499)

98: 920.04 0.550700 ( -0.143399 -0.287172 0.669080)

99: 926.96 2.919643 ( -0.688370 0.498906 -1.482189)

100: 929.87 0.177876 ( -0.405075 -0.109364 -0.042779)

101: 930.24 2.022034 ( 1.157466 -0.703553 0.432806)

102: 933.74 0.237071 ( 0.401667 0.246721 -0.121915)

103: 945.69 0.497433 ( 0.306792 -0.594755 0.222663)

104: 948.98 1.301556 ( -0.973857 0.568376 -0.173515)

105: 975.32 17.317631 ( -0.559106 4.075120 0.631212)

106: 996.22 2.337612 ( 0.729987 1.244434 0.506078)

107: 996.31 3.532123 ( 1.751841 -0.644418 0.218863)

108: 1010.64 15.029112 ( 2.355334 -3.068403 -0.257713)

109: 1023.44 13.253459 ( 1.839357 2.698982 1.608018)

110: 1029.02 0.221344 ( 0.162602 -0.129287 0.422124)

111: 1030.23 2.585282 ( -0.894890 1.318134 -0.216741)

112: 1039.15 4.998800 ( -1.081331 -0.848929 1.763191)

113: 1042.86 5.338558 ( 1.638429 -1.085661 1.214680)

114: 1073.66 7.913103 ( 0.710242 0.828074 -2.592866)

115: 1076.26 3.915941 ( 1.711159 -0.238863 -0.964791)

116: 1086.07 8.113493 ( 1.492986 -0.586315 -2.353874)

117: 1088.12 1.420285 ( -0.932372 -0.455147 0.586351)

118: 1094.15 12.445658 ( -3.500526 -0.026616 -0.437343)

119: 1133.63 23.959306 ( 4.388185 2.166884 0.088037)

120: 1141.35 20.547033 ( 3.964526 2.141604 -0.493047)

121: 1155.36 0.382980 ( 0.298571 0.536324 0.078683)

122: 1155.54 1.271605 ( -0.443170 -0.783511 0.679202)

123: 1157.57 0.500356 ( -0.613332 0.352290 -0.008417)

124: 1158.60 3.500876 ( 0.867643 -1.335475 -0.982130)

125: 1168.07 3.022545 ( 1.725924 -0.115326 -0.174442)

126: 1169.82 5.977008 ( -1.831043 1.553400 -0.459606)

127: 1172.46 1.917001 ( 0.462345 1.274897 0.279064)

128: 1180.35 8.129425 ( 2.015199 -1.954680 0.497619)

129: 1211.00 5.172055 ( 1.451562 -0.777069 1.568817)

130: 1241.94 38.646145 ( 3.710975 -4.980050 0.271873)

131: 1250.62 43.613499 ( 6.459578 -0.294271 -1.341924)

132: 1255.98 22.059151 ( -4.639661 0.289694 -0.669909)

133: 1276.85 12.496499 ( 2.637197 -0.005683 2.354073)

134: 1281.21 62.935809 ( -3.792848 6.823897 1.408736)

135: 1300.28 475.600318 (-19.515051 -8.382648 4.949172)

136: 1302.53 13.073785 ( -3.231662 0.157816 1.614075)

137: 1312.40 50.219813 ( -4.926445 2.161958 -4.612579)

138: 1315.10 29.680510 ( 1.871308 -3.819978 3.403892)

139: 1323.58 42.678490 ( -6.526930 -0.276951 -0.031167)

140: 1326.45 10.588506 ( -1.736651 -2.419426 1.311079)

141: 1334.84 1.257763 ( 0.833777 0.731655 -0.165103)

142: 1336.93 10.879238 ( 0.627719 -3.178256 0.619592)

143: 1340.96 3.395750 ( -0.842968 1.630430 -0.163867)

144: 1345.63 11.476108 ( -3.226025 0.169257 -1.019913)

145: 1418.37 20.878283 ( 4.482099 0.028092 0.887853)

146: 1421.72 11.862235 ( 2.456467 -1.999457 -1.352840)

147: 1429.24 3.103749 ( -0.102481 1.298678 1.186036)

148: 1429.27 10.661603 ( -2.298046 -1.800317 1.462685)

149: 1435.04 5.630205 ( 1.424164 -0.111794 -1.894588)

150: 1439.10 6.865082 ( 0.695567 -0.209678 2.517400)

151: 1461.94 18.207324 ( -3.085758 1.338256 2.625737)

152: 1465.30 3.165084 ( -0.720525 -1.065754 -1.228859)

153: 1481.74 4.844654 ( -2.118883 -0.566155 -0.185629)

154: 1482.66 1.924922 ( 1.173599 -0.712850 0.198572)

155: 1546.13 70.371441 ( -7.934472 2.723106 -0.017028)

156: 1559.58 4.427783 ( 1.771293 1.107018 0.254588)

157: 1568.59 14.314039 ( -3.626230 0.626937 0.878319)

158: 1572.68 2.769314 ( 1.350013 0.621514 -0.748666)

159: 1576.28 106.385366 ( -9.958590 0.820564 -2.557052)

160: 1588.21 4.507376 ( -1.047342 1.248366 1.360894)

161: 1589.69 11.658842 ( -1.558929 -2.939194 0.767936)

162: 1592.23 10.544441 ( 2.985443 0.080496 1.274790)

163: 1594.64 3.346240 ( -1.776156 -0.435806 -0.039806)

164: 1609.18 238.058331 ( 13.952399 6.055861 -2.591413)

165: 1613.92 182.381973 ( -2.832283 0.720150 13.184898)

166: 1615.92 19.348433 ( -1.726762 0.718243 -3.981313)

167: 1619.28 71.504504 ( 8.096383 1.859178 1.580046)

168: 1632.10 139.956543 ( -2.371537 -8.252467 8.138129)

169: 2939.21 43.473167 ( 3.905014 -3.464861 -4.027253)

170: 2976.81 13.350402 ( -1.732218 -2.795148 -1.592788)

171: 3011.42 12.793543 ( -1.094438 -2.457387 2.357329)

172: 3025.64 1.862353 ( 0.108099 1.111908 0.783791)

173: 3094.40 3.528683 ( -0.527748 1.774126 -0.320379)

174: 3096.14 2.558899 ( 0.996599 1.220742 0.274734)

175: 3103.57 0.412134 ( 0.500492 -0.360402 0.178192)

176: 3104.25 0.014665 ( -0.112503 -0.037097 -0.025145)

177: 3104.70 0.077047 ( 0.152491 0.080078 0.217672)

178: 3106.61 8.923465 ( -0.272501 -2.963612 -0.257318)

179: 3111.83 3.882408 ( 1.204300 1.415273 -0.655036)

180: 3112.90 1.029479 ( -0.525351 0.598387 -0.628823)

181: 3114.44 6.057487 ( -0.912331 -0.567823 2.214208)

182: 3119.54 8.689590 ( -1.010096 0.215007 2.760990)

183: 3119.97 2.566238 ( -0.628969 -1.046629 1.036920)

184: 3120.19 17.638941 ( 1.711470 -1.536008 -3.514326)

185: 3123.65 12.475908 ( 3.008298 -1.814655 0.364801)

186: 3128.76 8.728722 ( 1.366645 1.604773 2.070195)

187: 3128.86 19.296892 ( 1.827070 3.774133 -1.309438)

188: 3129.71 20.847287 ( -3.773887 1.528228 -2.066298)

189: 3132.90 6.718834 ( 1.178386 -1.430918 1.811826)

190: 3139.57 6.083181 ( -1.925835 0.916775 1.238493)

191: 3402.85 139.043387 ( 1.821580 -4.647982 10.682766)

192: 3409.39 243.837187 ( 15.077827 -3.935443 1.004294)

193: 3598.07 81.969802 ( 7.677301 3.846065 -2.869953)

194: 3709.90 16.613707 ( 2.021447 0.384779 3.518437)

**SI Table S11** IR frequencies for $\left[ Ni\left( EDA2BB \right)\left( OH_{2} \right)_{2} \right]$ complex in gas phase

The first frequency considered to be a vibration is 11

The total number of vibrations considered is 184

Mode freq (cm**-1) T**2 TX TY TZ

-------------------------------------------------------------------

11: 37.29 0.389800 ( 0.129375 0.534338 0.295880)

12: 47.32 0.871876 ( 0.555226 0.492541 -0.566571)

13: 51.96 2.648986 ( -0.835988 1.386403 0.167324)

14: 59.39 0.201243 ( -0.176765 0.218460 -0.349675)

15: 61.57 1.573728 ( -0.497595 0.081811 1.148666)

16: 64.01 1.528180 ( -0.663612 -0.482729 -0.924539)

17: 66.94 1.685813 ( 0.466114 0.368175 1.154555)

18: 71.46 0.301791 ( 0.149466 -0.257598 0.461622)

19: 84.42 3.408999 ( 1.271710 -0.659754 -1.164679)

20: 91.63 4.658534 ( 0.620140 -2.020431 0.437973)

21: 104.47 6.186659 ( -0.898536 -0.235263 2.307367)

22: 114.30 3.970902 ( 0.490261 -1.349901 1.381417)

23: 121.96 5.196191 ( -0.532541 1.869059 1.191306)

24: 130.84 7.383333 ( -0.456265 2.289699 1.390120)

25: 137.46 2.141202 ( 0.699946 -0.519803 -1.175195)

26: 139.77 36.175906 ( 2.052761 -5.614679 0.661405)

27: 144.52 1.612826 ( -0.250551 -1.244826 -0.021421)

28: 147.89 2.421243 ( -1.090750 0.245336 -1.082274)

29: 176.21 0.930184 ( 0.942576 -0.181999 0.092796)

30: 191.59 0.373338 ( 0.597177 -0.061114 -0.113942)

31: 197.87 1.859124 ( 0.362623 0.633750 -1.151516)

32: 215.20 0.967631 ( -0.410046 0.807279 0.384439)

33: 222.59 5.330018 ( -0.577236 0.503498 -2.177913)

34: 229.33 2.104028 ( 0.060904 -0.730651 1.251586)

35: 248.35 5.158111 ( -1.792248 -1.001482 0.971078)

36: 253.23 0.693919 ( -0.611287 -0.175949 -0.537857)

37: 277.96 5.911922 ( 1.920956 0.674457 1.329269)

38: 303.33 1.573872 ( -1.026529 -0.691504 0.204776)

39: 308.20 3.394210 ( 1.650433 0.514592 0.636771)

40: 310.24 4.195344 ( -1.084070 -1.185159 1.271038)

41: 317.21 22.431677 ( 0.883819 -0.699461 4.600141)

42: 334.82 6.110591 ( -2.165345 0.655728 -0.995938)

43: 343.93 2.249336 ( 1.488681 -0.091416 0.157508)

44: 351.93 6.692667 ( 0.017628 -0.600408 2.516320)

45: 377.58 4.939493 ( -1.767955 1.344400 0.080108)

46: 389.16 21.418553 ( 4.394542 1.450707 0.044745)

47: 392.83 1.835009 ( -1.339827 -0.165587 0.111603)

48: 396.52 3.264057 ( 1.805268 -0.011535 0.070217)

49: 410.46 9.209179 ( -1.120742 -1.600739 2.321799)

50: 412.48 33.898083 ( 5.297159 1.846408 1.558513)

51: 420.30 7.820749 ( -0.081582 0.109211 2.793236)

52: 437.04 18.373099 ( 1.454503 0.035164 4.031908)

53: 444.12 14.913835 ( 2.993247 0.276513 2.424428)

54: 457.23 41.075681 ( 1.409413 -0.113935 6.251100)

55: 470.83 88.276997 ( -4.238174 0.565983 8.366274)

56: 478.18 18.659656 ( 0.044127 0.606832 -4.276618)

57: 488.40 5.895771 ( -1.789857 0.460449 -1.574855)

58: 508.06 20.266961 ( -4.094946 -0.235358 1.855528)

59: 548.09 70.081195 ( 4.592538 -6.996313 -0.203447)

60: 553.44 33.158157 ( 0.639158 -5.676356 -0.727062)

61: 564.34 11.526475 ( -1.053122 3.171442 -0.599471)

62: 565.96 9.262806 ( -0.367903 2.427376 -1.798694)

63: 599.08 3.980124 ( -0.453640 1.568537 1.146310)

64: 611.33 0.616838 ( 0.627061 0.469954 0.052684)

65: 612.77 0.664382 ( -0.022415 -0.356850 -0.732487)

66: 632.93 43.417843 ( 0.553421 -1.619624 6.363049)

67: 635.32 109.708957 ( 0.788434 0.424311 10.435866)

68: 643.23 41.279755 ( -2.144494 -0.261847 6.050813)

69: 645.36 36.646105 ( -1.839392 2.411059 5.239231)

70: 664.02 41.227035 ( -0.865768 -1.022887 6.279425)

71: 677.63 3.448182 ( -1.447315 -0.042824 1.162595)

72: 681.83 2.820508 ( 0.415683 1.609896 0.236536)

73: 686.04 6.454275 ( 0.368881 -0.233045 2.502777)

74: 692.51 34.661828 ( 0.615654 2.987023 -5.035920)

75: 695.36 48.775013 ( 3.912478 -3.955149 4.221887)

76: 709.89 20.832237 ( 4.459193 -0.642440 0.731509)

77: 732.79 20.761957 ( 0.791338 2.217696 -3.900970)

78: 735.97 14.974773 ( 3.569705 0.624532 1.357180)

79: 749.22 45.329394 ( 6.106067 2.834785 -0.096605)

80: 749.77 17.167381 ( 0.275416 3.493751 -2.210256)

81: 761.57 1.066598 ( 0.983987 0.308871 0.054456)

82: 764.49 13.436827 ( 1.387794 0.696862 -3.320428)

83: 772.02 5.222485 ( 0.413277 0.755858 -2.116688)

84: 782.84 17.334788 ( 3.290469 -2.499425 -0.510369)

85: 785.50 6.967630 ( 0.199011 -0.767366 -2.517772)

86: 812.00 2.375615 ( 0.116181 0.760398 1.335631)

87: 827.80 3.125437 ( 0.209812 0.937988 -1.483777)

88: 827.94 13.406293 ( -1.964935 0.171930 3.084763)

89: 831.11 0.686351 ( 0.443006 0.571763 0.403959)

90: 850.60 1.256435 ( -0.435746 -0.431141 -0.938444)

91: 858.18 0.403643 ( 0.112074 0.306515 -0.545097)

92: 861.55 2.828985 ( -1.461680 -0.222560 0.801838)

93: 886.76 0.678413 ( 0.530568 -0.576135 0.254910)

94: 900.92 0.210548 ( -0.446566 0.057027 -0.088743)

95: 904.77 2.569019 ( -0.626351 1.308677 -0.681224)

96: 907.60 0.532358 ( 0.348483 -0.585171 0.261712)

97: 921.17 0.921627 ( -0.422035 -0.844834 0.172540)

98: 923.34 0.616338 ( -0.572390 0.472796 -0.255285)

99: 926.17 1.723613 ( 0.678862 -1.082062 -0.303153)

100: 930.55 0.036582 ( 0.160326 0.061559 0.084192)

101: 932.05 0.396396 ( 0.563553 0.110388 0.258106)

102: 938.06 0.282048 ( -0.395353 -0.218333 0.279418)

103: 945.61 0.235428 ( 0.265693 -0.366485 0.174712)

104: 946.81 0.363524 ( -0.244249 0.547031 -0.067997)

105: 980.14 12.030929 ( -0.553425 3.247234 1.086334)

106: 995.79 3.256264 ( 1.514407 0.853135 0.484764)

107: 996.30 2.064094 ( 0.649451 -1.257337 -0.247816)

108: 1009.51 2.014175 ( 1.245094 0.548476 0.403844)

109: 1023.48 16.086258 ( -1.680903 3.585207 0.638058)

110: 1028.04 2.004703 ( -1.125735 -0.584505 -0.629110)

111: 1032.56 1.406671 ( 0.818342 -0.844501 0.154289)

112: 1041.34 5.163714 ( 1.896562 -0.932669 0.834802)

113: 1045.19 5.776183 ( 0.538700 1.774639 -1.528608)

114: 1074.39 7.180046 ( 2.071787 0.928997 -1.422923)

115: 1077.32 1.922832 ( -0.773140 -0.917420 -0.695288)

116: 1078.33 4.136375 ( -1.869355 -0.381350 -0.704599)

117: 1092.42 7.845685 ( -2.545533 0.340333 -1.118088)

118: 1103.54 22.462119 ( -4.571020 -0.737870 1.011651)

119: 1134.09 55.202912 ( 7.094643 2.195276 -0.222988)

120: 1136.90 12.312111 ( 3.168931 0.895380 -1.211726)

121: 1155.68 0.228249 ( -0.433724 -0.163254 0.116105)

122: 1156.06 0.204221 ( 0.322845 0.261972 0.177095)

123: 1158.30 1.993965 ( 0.393161 1.331469 0.258030)

124: 1159.00 3.915929 ( 1.075035 -1.463964 -0.785519)

125: 1169.41 4.444167 ( -2.000600 -0.470583 0.469382)

126: 1169.72 1.771179 ( 1.197958 0.562190 0.141485)

127: 1173.08 3.000428 ( -0.554564 1.591523 -0.399927)

128: 1174.46 0.400470 ( 0.340414 0.220574 -0.485732)

129: 1211.71 1.242660 ( 0.920918 0.512957 -0.362555)

130: 1235.15 22.901716 ( 3.452435 -3.294949 -0.354566)

131: 1252.62 123.104934 (-11.083518 -0.430293 0.274627)

132: 1256.58 13.395604 ( 1.941467 1.757941 -2.556551)

133: 1283.53 87.386136 ( 4.755167 8.040960 0.342769)

134: 1283.89 50.626108 ( -7.070078 -0.048626 -0.798586)

135: 1286.12 211.330555 ( 14.388715 -1.272379 -1.635994)

136: 1301.16 0.906026 ( -0.250372 0.875835 0.276141)

137: 1306.07 117.514891 ( 8.511526 5.163303 -4.290586)

138: 1310.78 67.391272 ( -4.744720 -6.122542 2.719076)

139: 1318.75 40.450288 ( -4.879627 -3.684782 1.749832)

140: 1328.10 7.200825 ( -1.081989 -2.440489 0.272285)

141: 1333.37 1.041373 ( -0.343219 -0.894472 0.351416)

142: 1337.10 2.485074 ( 0.613423 -1.305309 0.636360)

143: 1340.44 4.934913 ( -1.514641 1.613868 -0.190275)

144: 1344.64 28.243703 ( -0.068023 -4.786060 2.309265)

145: 1427.20 7.301497 ( 2.430652 0.632311 0.996799)

146: 1430.53 3.460977 ( -0.948964 0.674450 1.451055)

147: 1430.71 3.654536 ( -0.025513 -1.803451 -0.633600)

148: 1435.03 6.158219 ( -1.894914 0.022755 1.602186)

149: 1436.43 8.798030 ( -2.608436 -1.332921 -0.466275)

150: 1440.37 1.713680 ( 1.110356 -0.658088 -0.218426)

151: 1461.61 14.519493 ( 2.096346 -1.741358 -2.663175)

152: 1467.83 9.967309 ( 1.127499 2.768483 1.015655)

153: 1481.22 4.335103 ( 1.692666 0.747160 0.954850)

154: 1483.02 3.303319 ( -1.683800 0.236117 0.642173)

155: 1528.52 36.484556 ( 4.866538 -3.260265 1.473784)

156: 1554.28 16.941341 ( -1.637935 -3.677048 0.858968)

157: 1561.09 7.073739 ( -1.871000 -0.331650 -1.860942)

158: 1572.46 0.969005 ( 0.722227 0.661830 0.096818)

159: 1572.67 1.551552 ( -0.233459 -0.452431 1.136818)

160: 1587.29 22.942283 ( 2.695527 3.590846 -1.668006)

161: 1589.25 5.692153 ( -1.035514 1.809386 -1.160166)

162: 1593.39 2.723204 ( -0.157079 -1.277608 -1.032593)

163: 1594.18 1.671952 ( 1.203435 0.463542 0.093939)

164: 1605.80 15.875350 ( -2.072931 -0.679559 3.334143)

165: 1619.51 186.142685 ( 10.318061 6.767508 -5.820751)

166: 1629.99 35.829488 ( 3.429434 -4.743660 -1.251463)

167: 1636.59 318.512680 (-14.189384 -8.839422 -6.248094)

168: 1645.56 310.904577 ( 4.545460 1.974436 -16.921731)

169: 2974.81 20.871330 ( 2.875893 -2.933070 -1.999418)

170: 2991.71 13.096693 ( 1.096834 3.335749 0.875456)

171: 3033.34 12.031962 ( -0.331248 -1.564175 3.078245)

172: 3045.79 10.238597 ( -1.033038 2.953570 -0.669219)

173: 3095.72 2.328415 ( 0.837916 1.192537 0.451850)

174: 3099.91 2.349333 ( 1.390004 -0.645483 0.023970)

175: 3103.64 0.198801 ( -0.145849 -0.120168 -0.403842)

176: 3105.03 0.040788 ( 0.065021 0.038648 0.187262)

177: 3106.50 0.333866 ( 0.244604 -0.483587 -0.200447)

178: 3107.61 0.620265 ( 0.591924 -0.487154 -0.180476)

179: 3112.12 3.931638 ( -1.380559 -1.406762 0.216139)

180: 3113.34 8.192883 ( 0.158981 -2.847946 0.238354)

181: 3115.00 6.136828 ( 2.423948 -0.128526 0.494759)

182: 3116.09 1.668407 ( 0.718222 -0.725255 0.791562)

183: 3119.97 9.231357 ( -2.048217 0.375854 2.212441)

184: 3122.45 9.091752 ( -1.293600 -2.367089 -1.347309)

185: 3123.79 6.082510 ( -0.964914 1.791266 1.393850)

186: 3124.84 12.471020 ( -1.721114 -0.238413 3.074402)

187: 3128.92 12.882954 ( -1.621180 -2.907183 -1.342764)

188: 3130.10 12.833737 ( -2.773877 2.204615 0.528222)

189: 3131.40 13.428502 ( 2.672811 -1.251370 2.172247)

190: 3134.42 7.070917 ( -0.967104 -2.024791 1.426832)

191: 3467.93 196.066184 (-10.221221 4.726689 -8.321733)

192: 3501.31 106.919463 ( 1.361960 6.638507 -7.809914)

193: 3650.46 50.439186 ( 6.433161 2.997495 -0.262018)

194: 3721.75 12.914607 ( -2.174607 0.292575 -2.846066)

**SI Table S12** IR frequencies for $\left[ Mn\left( EDA2BB \right)\left( OH_{2} \right)_{2} \right]$ complex in gas phase

The first frequency considered to be a vibration is 9

The total number of vibrations considered is 186

Mode freq (cm**-1) T**2 TX TY TZ

-------------------------------------------------------------------

9: 40.17 0.433174 ( -0.468418 -0.414657 -0.204495)

10: 45.52 0.865692 ( -0.467259 0.795797 -0.118611)

11: 49.64 0.156147 ( 0.113971 -0.375996 -0.042245)

12: 59.99 1.088576 ( -0.988484 0.213043 -0.257078)

13: 62.17 1.125152 ( 0.080373 0.976042 0.407472)

14: 77.72 1.029408 ( 0.376662 -0.750145 -0.569927)

15: 80.96 1.441366 ( 0.910959 -0.777280 -0.085763)

16: 88.70 1.199041 ( 0.210739 -0.933003 0.533044)

17: 108.39 0.563603 ( 0.746867 0.063956 -0.041251)

18: 113.38 5.391273 ( -0.300536 0.681597 2.199176)

19: 119.78 2.519105 ( 0.012109 -0.310725 1.556409)

20: 131.04 0.335444 ( 0.108587 -0.368749 0.433217)

21: 132.99 4.256093 ( 0.648798 -1.413663 1.355253)

22: 149.53 1.597102 ( 0.174439 0.807626 -0.956250)

23: 157.24 0.072142 ( -0.255042 0.044811 0.071330)

24: 173.98 11.958538 ( -2.569982 -1.291449 1.919867)

25: 174.99 4.343936 ( -1.577954 -1.273293 -0.482415)

26: 187.67 1.317577 ( -0.344774 0.886191 0.642942)

27: 197.01 0.648047 ( -0.476160 0.443560 -0.473891)

28: 210.95 5.811206 ( -1.592301 1.575338 -0.891120)

29: 215.13 7.931872 ( -1.390024 0.198804 2.441349)

30: 227.08 18.636449 ( -1.472216 -0.902151 3.956659)

31: 239.42 4.873325 ( -1.136019 -1.425555 -1.245222)

32: 243.84 8.529716 ( -2.204579 -1.512141 1.175999)

33: 247.10 16.782721 ( 0.578971 -2.646925 -3.072670)

34: 265.19 4.481646 ( -1.843416 -1.030907 0.143859)

35: 271.13 6.528416 ( -1.729922 1.408058 1.246258)

36: 278.57 1.676073 ( 1.256996 -0.003069 0.309879)

37: 285.08 4.150642 ( -0.796069 0.655752 -1.756959)

38: 296.58 12.780325 ( 1.924501 -2.157930 -2.102369)

39: 305.15 11.697823 ( 2.801452 1.896572 0.502695)

40: 323.10 12.889538 ( -1.898015 2.816431 1.163956)

41: 339.94 15.409725 ( 3.861090 -0.633524 0.316796)

42: 357.47 3.230938 ( -0.811063 -1.492327 -0.588281)

43: 362.17 3.451912 ( 1.685848 0.113747 0.772587)

44: 384.83 12.856191 ( -3.006827 -1.919509 -0.361481)

45: 387.37 10.347895 ( 1.718384 1.713426 2.111687)

46: 395.39 0.176993 ( 0.202971 -0.091165 0.357049)

47: 399.00 2.164728 ( -0.965266 0.339753 1.057146)

48: 402.90 1.946109 ( 0.718103 -0.246974 1.170231)

49: 429.55 15.494789 ( -3.171479 2.326319 0.157337)

50: 437.71 19.553201 ( -1.682990 1.107075 -3.936385)

51: 450.20 1.241500 ( -0.948378 0.336825 0.478151)

52: 459.43 9.165311 ( -2.578132 -1.500807 -0.515872)

53: 473.44 22.240613 ( 4.588654 -1.057935 0.256197)

54: 495.91 2.352787 ( -1.499558 -0.139789 -0.290814)

55: 532.29 84.248615 ( -8.801831 1.546050 -2.094307)

56: 561.60 12.033430 ( 0.168786 -2.555961 2.339232)

57: 566.34 21.748387 ( 4.640088 -0.215341 0.414249)

58: 569.46 3.937407 ( 1.939655 -0.281913 0.309308)

59: 580.63 45.208157 ( -0.204824 -5.604279 3.709213)

60: 593.36 3.942835 ( -1.890881 -0.346599 0.497265)

61: 609.83 0.132753 ( -0.353253 -0.083214 0.032262)

62: 614.33 1.134775 ( -0.820582 0.146957 -0.663192)

63: 618.68 12.902046 ( -3.293774 -0.457867 -1.357740)

64: 633.19 26.465482 ( -3.595518 3.530465 -1.036123)

65: 640.50 11.539876 ( -2.855116 -1.675664 0.761798)

66: 648.59 0.954133 ( 0.238140 0.895475 -0.309106)

67: 653.37 1.534906 ( 0.202985 -1.203916 -0.210448)

68: 661.06 15.404665 ( -2.368594 -0.778453 -3.031244)

69: 678.68 26.909603 ( -2.867763 -3.685580 -2.258769)

70: 683.63 14.950850 ( 0.975928 2.576923 2.712542)

71: 693.84 37.105711 ( -4.481763 3.634732 -1.951470)

72: 717.64 23.743410 ( -4.843397 0.143052 0.514245)

73: 725.23 33.970217 ( -0.006740 4.254088 -3.984082)

74: 726.33 34.330105 ( 5.343977 2.105682 -1.156769)

75: 736.20 78.965408 ( 1.583445 -7.750491 4.048210)

76: 742.59 11.024420 ( 2.857899 1.484619 -0.807923)

77: 744.27 2.174525 ( 0.298168 0.059684 1.442934)

78: 751.29 42.274897 ( -1.704840 3.680324 -5.081696)

79: 757.44 18.724586 ( -1.569643 -1.641553 -3.683220)

80: 770.31 26.006141 ( 2.915564 3.087377 -2.823780)

81: 774.60 23.811652 ( 0.602604 1.908212 -4.450533)

82: 778.18 20.283364 ( -3.702184 1.034716 2.346606)

83: 787.81 150.210381 ( -2.438282 -6.661595 9.994414)

84: 804.28 35.590702 ( 2.751565 -0.717746 5.244467)

85: 815.24 0.403667 ( -0.400269 0.046353 0.491226)

86: 821.09 28.131073 ( -0.740421 -0.315872 -5.242430)

87: 827.41 0.152931 ( -0.340038 -0.000994 0.193144)

88: 850.01 3.089292 ( 1.665963 0.090187 -0.552925)

89: 854.33 0.205685 ( -0.316014 0.036739 0.323219)

90: 862.53 1.170488 ( -0.215413 0.905999 -0.550682)

91: 880.17 0.967054 ( -0.087146 -0.413651 -0.887892)

92: 888.42 0.666767 ( 0.775787 -0.168306 0.191299)

93: 896.78 2.800361 ( 0.777571 1.476830 -0.121321)

94: 912.50 0.953529 ( -0.250703 -0.655879 0.678602)

95: 913.62 0.828026 ( -0.870898 0.220138 -0.145261)

96: 919.24 0.419179 ( 0.219376 -0.170137 0.584899)

97: 921.14 3.798207 ( -0.414263 0.548924 -1.823534)

98: 923.06 0.101015 ( -0.245326 -0.010703 -0.201782)

99: 924.42 0.591632 ( 0.513998 0.183305 -0.542067)

100: 925.98 0.185092 ( 0.359706 0.235681 -0.012555)

101: 935.80 0.085282 ( 0.248545 0.093416 -0.121577)

102: 940.74 0.624417 ( 0.338537 0.634768 -0.326924)

103: 976.66 15.762574 ( 0.625157 3.827464 0.849866)

104: 991.99 0.898780 ( 0.901195 -0.244377 -0.164034)

105: 996.71 4.444833 ( 1.829440 0.907525 0.523813)

106: 1016.52 6.668660 ( -1.802327 -1.439264 1.161376)

107: 1024.96 7.962247 ( 1.476074 1.433777 1.930734)

108: 1028.45 1.213069 ( -0.231222 0.763959 -0.758928)

109: 1031.51 8.217770 ( -2.384741 1.395723 0.763373)

110: 1038.07 10.944330 ( 2.389387 1.679968 -1.553341)

111: 1045.24 8.287990 ( 2.357281 -1.471555 -0.752159)

112: 1059.48 91.854052 ( 1.903119 -3.070342 8.877229)

113: 1067.48 1.854092 ( 1.275586 -0.344088 -0.329507)

114: 1073.49 4.890312 ( 1.126348 0.894523 -1.679726)

115: 1083.12 8.922937 ( -2.067341 -1.019376 1.899976)

116: 1088.91 1.183643 ( -0.464232 0.255239 0.950255)

117: 1094.81 10.433660 ( 3.170712 -0.571972 0.230424)

118: 1124.33 95.670936 ( 8.138450 5.383159 -0.676882)

119: 1140.89 30.538778 ( 5.218413 0.370457 -1.780368)

120: 1153.00 0.598254 ( -0.432276 -0.638902 -0.056534)

121: 1153.36 1.568212 ( -1.129090 -0.253453 0.478674)

122: 1154.23 0.241651 ( -0.137526 0.279200 0.380507)

123: 1154.69 0.050274 ( -0.076502 0.206440 -0.042479)

124: 1160.68 11.076893 ( 3.138388 1.095106 0.167799)

125: 1162.28 5.545967 ( -2.259249 -0.544860 0.380641)

126: 1170.16 1.683189 ( -1.148671 -0.491739 -0.349196)

127: 1179.19 1.568582 ( -0.497958 -0.876960 0.742672)

128: 1202.89 2.087343 ( -0.183228 -0.341715 1.391762)

129: 1235.41 8.810199 ( 2.700975 1.142532 0.457769)

130: 1241.53 126.399434 ( 9.954767 3.798500 -3.587959)

131: 1249.98 11.650843 ( -2.415482 2.107834 1.171891)

132: 1270.94 148.673825 ( 9.849150 6.383336 -3.304707)

133: 1273.56 18.656819 ( -1.418000 -2.987569 -2.778584)

134: 1276.47 186.703554 ( 10.849211 7.066432 -4.366203)

135: 1297.12 2.125564 ( 0.602768 -0.647748 -1.158731)

136: 1305.55 64.449782 ( 6.925588 0.329725 -4.046888)

137: 1310.57 11.712441 ( 3.001713 -1.568403 -0.492213)

138: 1315.18 40.252819 ( -6.299484 -0.651992 0.379769)

139: 1324.51 12.083325 ( 2.624516 2.199954 0.596189)

140: 1329.92 1.026034 ( 0.290135 0.003954 0.970485)

141: 1331.20 1.677119 ( -0.298561 0.488205 -1.161738)

142: 1338.33 7.892213 ( -1.698551 -2.011123 0.981081)

143: 1338.54 0.434465 ( -0.540494 -0.112452 0.360120)

144: 1398.26 247.041807 ( 15.629445 0.316230 1.631644)

145: 1423.99 25.970328 ( 1.174883 -3.880909 -3.086830)

146: 1426.50 6.501327 ( -0.054441 -2.320934 1.054338)

147: 1430.16 16.591399 ( -3.610819 -0.657348 1.766714)

148: 1433.19 37.942679 ( 6.119597 0.289704 -0.639752)

149: 1434.64 56.734507 ( -7.376412 0.742159 -1.331260)

150: 1436.23 10.076600 ( -2.055977 -0.725231 -2.307293)

151: 1463.16 12.451460 ( 2.277581 0.124787 -2.692306)

152: 1468.06 6.939263 ( -1.782108 1.617726 1.070661)

153: 1478.53 22.017693 ( 4.224854 -1.963496 0.559453)

154: 1481.23 15.588503 ( -3.430617 -1.621218 -1.091340)

155: 1492.23 129.948454 ( 9.114103 -6.816611 -0.644508)

156: 1547.41 1.247563 ( 0.459153 -0.269899 -0.981783)

157: 1559.78 2.691752 ( -0.698855 1.187852 0.890147)

158: 1564.67 5.007529 ( 1.936760 -0.724554 -0.855285)

159: 1570.23 14.559949 ( -1.527234 -0.395445 3.474353)

160: 1572.78 179.564461 ( 4.236926 -0.032600 -12.712665)

161: 1586.50 6.103897 ( 2.464865 0.073179 0.151599)

162: 1587.43 42.257776 ( 0.044982 0.181086 6.497920)

163: 1589.88 14.708247 ( -3.300062 1.675642 1.005018)

164: 1592.19 11.453165 ( 2.646231 1.671334 1.287349)

165: 1596.03 34.011943 ( 5.644153 0.965938 1.105641)

166: 1609.22 66.527229 ( -0.688388 -3.567021 7.302719)

167: 1652.60 529.594824 (-16.727404 -12.751348 -9.337660)

168: 1870.66 12.658999 ( 2.178172 2.220862 -1.726945)

169: 2928.04 450.355035 ( 19.643277 -7.473054 -2.941116)

170: 2980.24 29.113894 ( 3.934123 3.131689 1.956807)

171: 2985.61 14.339500 ( 0.567529 -3.721006 0.414156)

172: 3022.97 3.584443 ( -0.013164 1.035937 -1.584646)

173: 3043.81 5.351762 ( 0.896766 0.559485 2.057802)

174: 3089.22 8.886405 ( -1.922491 -2.205747 -0.570188)

175: 3092.25 2.927488 ( -0.026523 -1.592556 0.624940)

176: 3100.78 6.307858 ( -2.248946 1.111332 0.122643)

177: 3101.55 0.287925 ( 0.292650 0.438395 -0.100448)

178: 3103.59 3.082480 ( 1.599067 0.233637 0.686207)

179: 3104.61 7.536432 ( -0.164655 2.738545 0.098449)

180: 3109.26 10.458681 ( -0.945091 -1.345612 2.784746)

181: 3109.71 4.329954 ( 1.202034 1.596921 -0.578714)

182: 3112.48 5.147795 ( 1.138346 0.047694 1.962062)

183: 3116.87 7.898488 ( -0.213254 0.679798 2.718618)

184: 3117.38 23.460719 ( 2.028397 -0.032879 -4.398323)

185: 3123.07 21.342884 ( 3.890584 -2.454367 0.426997)

186: 3123.81 17.242607 ( -2.967368 2.455396 1.551890)

187: 3126.14 11.633470 ( -0.950570 -2.664901 1.904781)

188: 3127.17 15.343966 ( 2.232617 2.889440 1.417929)

189: 3132.27 17.782236 ( -3.719987 1.346701 -1.459565)

190: 3134.60 10.088861 ( -0.701276 0.755097 -3.004480)

191: 3138.28 6.061850 ( -2.174313 0.884243 0.743187)

192: 3408.21 70.996241 ( -5.684327 2.411065 5.733362)

193: 3498.41 55.496132 ( -5.027727 -5.415328 -0.944622)

194: 3669.36 25.919431 ( 0.985815 2.762080 4.161552)

**SI Table S13** IR frequencies for $\left[ Co\left( EDA2BB \right)\left( OH_{2} \right)_{2} \right]$ complex in gas phase

The first frequency considered to be a vibration is 9

The total number of vibrations considered is 186

Mode freq (cm**-1) T**2 TX TY TZ

-------------------------------------------------------------------

9: 35.28 0.392363 ( 0.206766 0.591236 0.007067)

10: 44.47 0.236155 ( 0.350910 0.172780 0.288383)

11: 47.52 0.871704 ( 0.224329 -0.894223 -0.147464)

12: 60.61 1.455229 ( 1.175205 -0.218678 -0.162181)

13: 61.34 1.177100 ( 0.275315 -1.044017 -0.106440)

14: 72.12 0.305583 ( -0.191671 0.517109 0.037990)

15: 79.37 0.987779 ( -0.456236 0.843541 -0.260895)

16: 90.79 0.067333 ( 0.113074 -0.187256 -0.139580)

17: 101.94 0.753916 ( 0.707836 -0.108167 0.491105)

18: 107.36 1.681394 ( -0.966771 0.151496 0.850762)

19: 112.82 6.111364 ( -0.806137 1.202717 2.003742)

20: 127.05 7.797208 ( -0.094140 -1.700756 2.212640)

21: 130.68 5.159042 ( -0.066450 1.277398 -1.876934)

22: 136.18 3.078434 ( 0.490942 0.670298 -1.545351)

23: 149.25 6.215546 ( -1.201865 -0.372889 2.152213)

24: 153.39 2.130585 ( -0.394878 -1.103451 0.870088)

25: 173.53 4.958821 ( -0.968820 0.840637 -1.820313)

26: 174.85 0.307629 ( -0.510841 0.126779 -0.174921)

27: 177.19 5.346678 ( 1.500468 1.393157 -1.074424)

28: 191.10 5.263080 ( 0.353941 -0.597865 2.186404)

29: 198.55 0.422851 ( -0.049359 0.096488 0.641175)

30: 210.03 1.727007 ( -0.387169 0.437360 1.177210)

31: 218.17 2.365775 ( 0.256671 -0.895414 1.223981)

32: 234.98 5.808667 ( -1.100302 0.655380 -2.041685)

33: 244.72 1.800734 ( -0.504192 1.222177 -0.229802)

34: 255.23 10.498836 ( -3.014672 -1.138716 0.337519)

35: 259.14 1.957009 ( 0.770405 0.357050 -1.111756)

36: 278.24 6.366574 ( -1.409752 -0.662205 -1.985109)

37: 283.76 3.572112 ( -1.419758 -0.950606 0.807927)

38: 290.34 15.920529 ( -1.137084 2.350891 3.016766)

39: 309.23 5.293562 ( 1.401417 0.047525 -1.824098)

40: 316.47 9.106676 ( -2.153063 -0.884979 -1.920367)

41: 337.26 5.491551 ( -2.259779 0.118617 -0.608999)

42: 344.57 8.253470 ( -2.052105 0.976871 1.757287)

43: 360.50 6.648708 ( 1.803893 -1.242074 -1.360857)

44: 372.91 20.966772 ( 4.421080 -1.188195 -0.094968)

45: 384.29 5.356788 ( -0.898907 -1.936211 -0.894339)

46: 392.96 0.234645 ( -0.111772 -0.186001 0.433077)

47: 394.72 1.821590 ( -1.245944 0.041987 -0.517157)

48: 398.73 4.406969 ( 1.071418 0.562554 -1.715390)

49: 415.42 8.935219 ( 2.129848 -1.608189 -1.346363)

50: 427.31 29.061122 ( 1.388502 -2.561381 4.535693)

51: 442.34 49.403341 ( -1.815785 -5.352818 4.177751)

52: 445.11 10.535820 ( 1.050493 -2.978445 -0.749102)

53: 461.37 26.526641 ( -1.499746 4.927148 0.024830)

54: 470.04 6.294642 ( -2.286774 0.891797 0.519618)

55: 478.66 0.667427 ( -0.553825 -0.599801 0.030724)

56: 495.68 8.550948 ( -2.194121 -1.644069 1.016768)

57: 505.02 42.997819 ( 6.513897 0.606720 -0.445930)

58: 541.15 35.377807 ( -5.787546 0.075144 -1.369842)

59: 563.49 1.852196 ( -0.224004 -0.598919 1.201380)

60: 567.99 0.181688 ( 0.161182 0.383357 -0.093521)

61: 597.31 6.573767 ( 2.307091 -1.118064 0.032139)

62: 612.08 0.327790 ( 0.420932 0.291960 -0.255667)

63: 614.54 0.682271 ( -0.360085 0.130747 -0.731789)

64: 624.97 9.763482 ( -2.371609 -2.033981 -0.043293)

65: 638.63 16.474477 ( -3.792908 1.342477 -0.534863)

66: 645.72 5.073765 ( -1.510790 -1.636648 0.335652)

67: 656.88 8.623081 ( 1.004345 2.666665 0.709415)

68: 662.36 145.938890 ( 1.002536 1.718642 -11.915540)

69: 671.32 7.064162 ( 1.909013 1.659647 0.815723)

70: 674.59 0.354937 ( 0.273958 0.246953 0.467865)

71: 681.70 52.804614 ( -3.749899 -6.055103 -1.441734)

72: 687.24 167.602796 ( 0.962123 9.226001 -9.030948)

73: 691.78 25.831443 ( -4.061882 -1.760859 2.496384)

74: 694.88 39.458272 ( -4.892265 3.366669 -2.046840)

75: 718.01 16.239553 ( 3.839345 -0.565257 1.086033)

76: 729.36 19.350537 ( -3.489905 2.607144 -0.611474)

77: 733.45 34.366583 ( -3.036236 2.503658 -4.345060)

78: 742.16 10.697555 ( 1.582451 2.801905 -0.585433)

79: 747.19 35.125570 ( -3.723513 -3.961582 2.359425)

80: 757.98 7.549521 ( -0.364824 0.567857 -2.663449)

81: 762.14 16.692236 ( 3.293148 2.084786 1.225185)

82: 767.29 41.426833 ( -3.803754 3.329733 -3.983863)

83: 771.28 1.749211 ( 0.708872 -0.199218 -1.098646)

84: 779.72 1.602659 ( -0.404077 0.987209 0.681761)

85: 801.97 51.229072 ( 0.847789 -0.113008 7.106163)

86: 819.29 1.414344 ( 1.082383 -0.345042 -0.351763)

87: 827.12 24.254833 ( 0.842963 0.924054 -4.763441)

88: 830.08 0.120076 ( 0.033933 -0.332796 -0.090393)

89: 848.13 0.496150 ( -0.304820 -0.632163 -0.060037)

90: 857.22 0.434237 ( -0.625451 0.088384 0.187712)

91: 861.96 1.367775 ( 1.134904 0.251552 0.128415)

92: 867.69 74.947065 ( 0.841661 -1.724525 8.441841)

93: 884.81 2.198243 ( -0.138175 -0.897923 1.171702)

94: 895.97 0.608856 ( -0.329196 0.075276 0.703434)

95: 902.61 2.614329 ( -0.809149 1.381930 -0.223331)

96: 910.40 1.203796 ( 1.038785 -0.251864 0.247559)

97: 917.29 1.173018 ( 0.229611 1.057646 -0.041014)

98: 918.12 0.491086 ( -0.000731 -0.260562 0.650533)

99: 924.23 1.973640 ( -0.357435 0.576085 -1.230449)

100: 927.28 1.518495 ( -1.052172 0.632844 -0.104578)

101: 928.47 0.119461 ( 0.137239 0.022077 -0.316447)

102: 929.26 0.132190 ( -0.168658 -0.309334 -0.089760)

103: 943.27 0.459363 ( -0.535863 0.166342 -0.380190)

104: 944.46 0.358216 ( 0.234830 -0.485818 0.258943)

105: 987.14 13.854046 ( 1.156264 -3.529031 -0.251085)

106: 995.92 1.265914 ( -1.056146 0.358789 -0.147446)

107: 996.48 3.426278 ( -1.450929 -1.039895 -0.489592)

108: 1019.50 1.296139 ( 0.211376 -1.038307 0.416388)

109: 1026.43 7.326344 ( 0.778075 2.247862 1.291534)

110: 1030.49 1.852923 ( -0.794120 0.896877 -0.646459)

111: 1032.51 4.395064 ( 1.721348 -1.190600 -0.120410)

112: 1040.18 6.412268 ( 1.861822 1.171650 -1.254243)

113: 1043.80 5.551985 ( -1.405072 1.418982 -1.250699)

114: 1072.64 10.361814 ( -1.878204 -0.750193 2.504272)

115: 1074.98 1.152955 ( 0.298252 -0.121302 -1.024347)

116: 1086.99 7.977004 ( 1.427350 0.043689 -2.436754)

117: 1090.50 0.914833 ( -0.120712 -0.151033 0.936724)

118: 1097.07 19.598300 ( 4.423919 -0.164685 -0.010833)

119: 1134.92 43.156827 ( -5.741161 -3.139712 -0.581465)

120: 1142.90 22.324057 ( -4.428109 -0.877357 1.395045)

121: 1154.15 2.517667 ( 0.947954 1.186958 -0.458455)

122: 1154.99 0.270835 ( 0.063305 0.202028 0.475408)

123: 1156.40 0.038241 ( -0.024498 -0.009881 0.193760)

124: 1158.94 1.961581 ( -0.034763 1.260598 0.609315)

125: 1166.11 0.810560 ( 0.743393 -0.498321 0.097990)

126: 1167.15 2.300639 ( 1.191808 0.933782 -0.091017)

127: 1171.59 1.992912 ( 0.732307 1.193386 0.180186)

128: 1180.24 4.463925 ( 1.181086 -1.603731 0.704988)

129: 1208.39 8.426078 ( -2.215979 1.031194 -1.565935)

130: 1238.33 15.214664 ( 2.592857 -2.913421 0.061099)

131: 1248.81 55.604087 ( 7.069998 1.578950 -1.768087)

132: 1256.06 18.363289 ( -4.148960 1.036955 -0.272298)

133: 1277.78 9.659307 ( 2.799735 0.549717 1.232316)

134: 1279.89 36.701846 ( -2.580205 5.130556 1.929193)

135: 1296.90 419.959268 (-17.198649 -9.684575 5.511328)

136: 1300.24 4.231575 ( -1.690840 0.437320 1.086916)

137: 1311.53 75.636844 ( -8.688469 -0.205021 0.324517)

138: 1312.65 24.713988 ( -2.095648 3.700335 -2.574835)

139: 1321.03 7.770643 ( -2.728897 -0.563628 -0.078018)

140: 1328.44 4.836861 ( -0.034879 -2.145340 0.482866)

141: 1332.45 0.497554 ( -0.139284 0.546672 0.423443)

142: 1333.52 2.566970 ( 1.023747 0.116650 -1.226908)

143: 1338.94 4.043549 ( -0.781221 -1.843647 -0.184952)

144: 1341.40 7.282609 ( -0.316301 2.416671 -1.158560)

145: 1423.26 2.485512 ( -1.227717 -0.013512 -0.988959)

146: 1428.32 6.792346 ( 1.136524 1.822013 -1.476797)

147: 1428.98 6.837131 ( -0.396177 2.038080 1.589466)

148: 1432.52 4.716455 ( -1.860034 0.936844 0.615673)

149: 1434.71 6.479113 ( 1.814354 0.186794 -1.775483)

150: 1437.67 5.924188 ( -1.253410 0.169659 2.079511)

151: 1460.73 9.822564 ( -0.710365 -1.105280 -2.845400)

152: 1467.80 2.895074 ( 0.637327 1.077250 1.152571)

153: 1480.16 2.634417 ( 1.010566 0.803695 0.983488)

154: 1482.17 7.521495 ( -2.267815 1.535264 -0.146536)

155: 1506.34 11.815056 ( -2.265340 -2.575924 -0.218868)

156: 1551.30 11.056127 ( -1.206077 3.092375 -0.196778)

157: 1556.44 0.971956 ( 0.454518 -0.106879 -0.868301)

158: 1563.90 1.312775 ( 0.472003 0.433527 0.949759)

159: 1572.12 1.926438 ( -0.526369 -0.387841 1.224317)

160: 1576.57 6.126957 ( 1.714122 -1.742006 -0.392630)

161: 1587.78 16.033583 ( -1.216997 -1.876761 3.321185)

162: 1588.90 5.551608 ( -0.661879 -1.872658 -1.267547)

163: 1593.90 3.902746 ( -1.663434 -1.026231 -0.287377)

164: 1595.11 1.062139 ( 0.130182 0.202192 -1.002153)

165: 1601.76 181.683560 ( 9.790873 -0.030677 -9.263985)

166: 1606.83 25.858896 ( -4.088400 -1.690990 -2.506878)

167: 1624.64 177.837844 ( 5.057779 6.484051 -10.498276)

168: 1644.31 426.059399 ( 14.058012 10.255073 11.102485)

169: 2986.27 11.693702 ( -2.284849 -2.243200 -1.200509)

170: 2989.80 20.823521 ( 1.920263 -4.056707 -0.824162)

171: 3028.54 3.544791 ( 0.507272 0.388398 -1.771049)

172: 3052.23 1.546295 ( 0.570047 0.869386 0.682284)

173: 3093.58 3.583564 ( -1.118905 -1.512096 -0.212561)

174: 3095.11 2.308509 ( -0.289989 1.399393 -0.515862)

175: 3103.03 1.661901 ( 1.195753 -0.462644 -0.134303)

176: 3103.09 0.090961 ( 0.063503 0.123192 -0.267866)

177: 3104.15 0.113125 ( 0.058914 0.283856 0.170528)

178: 3106.09 9.870602 ( 0.133445 3.138856 0.019478)

179: 3110.34 4.022474 ( 1.228813 1.558760 -0.287678)

180: 3112.33 8.221127 ( 0.554375 0.778582 -2.703258)

181: 3113.36 1.960527 ( 0.897498 -0.356903 1.013729)

182: 3118.72 8.578557 ( -1.487799 0.139004 2.519065)

183: 3118.97 16.450605 ( -1.048064 0.555240 3.878644)

184: 3121.38 7.594417 ( 0.934430 -2.078878 -1.549039)

185: 3122.55 14.932633 ( -3.322348 1.770070 0.872636)

186: 3127.66 16.665831 ( -2.224361 -3.224867 -1.148164)

187: 3127.83 11.269333 ( 1.269450 2.449433 -1.912618)

188: 3130.04 19.369963 ( -3.376267 1.775029 -2.195463)

189: 3130.68 13.531790 ( 2.264171 -1.862223 2.222036)

190: 3147.29 7.383572 ( -1.810497 0.955712 1.786698)

191: 3280.30 288.311950 ( 16.018148 -5.535145 1.045485)

192: 3379.23 116.768057 ( -6.853265 0.575460 8.334846)

193: 3543.31 93.560612 ( -8.356010 -4.848343 0.480915)

194: 3700.51 14.257176 ( 1.396582 0.821620 3.410524)

**SI Table S14** IR frequencies for $\boldsymbol{EDA}\mathbf{2}\boldsymbol{BB}$ ligand in gas phase

The first frequency considered to be a vibration is 10

The total number of vibrations considered is 170

Mode freq (cm**-1) T**2 TX TY TZ

-------------------------------------------------------------------

10: 39.82 0.094762 ( 0.006529 0.303358 -0.051900)

11: 41.26 0.767633 ( -0.341240 -0.011113 0.806886)

12: 57.24 0.200121 ( -0.395242 0.001962 -0.209526)

13: 59.68 0.350668 ( 0.013343 -0.591600 -0.022357)

14: 67.86 0.290578 ( -0.300472 -0.037526 0.445966)

15: 76.24 0.255547 ( 0.026622 -0.504608 -0.014439)

16: 84.36 0.568991 ( 0.470101 0.025238 -0.589372)

17: 99.52 0.006917 ( -0.007990 0.079739 0.022254)

18: 112.65 0.003081 ( -0.006025 0.050294 -0.022708)

19: 119.44 1.413658 ( 0.188548 -0.008836 1.173895)

20: 126.11 0.601796 ( -0.035498 0.774642 0.021589)

21: 165.05 0.492807 ( -0.014457 0.701852 0.001632)

22: 171.92 0.232581 ( 0.012962 -0.482040 -0.007096)

23: 173.67 2.034143 ( 1.087952 0.048636 -0.920944)

24: 178.29 8.448994 ( 2.851408 0.157034 -0.542040)

25: 181.46 0.006676 ( -0.071442 0.037505 0.012865)

26: 215.70 0.292619 ( -0.032080 0.539976 -0.004003)

27: 221.65 1.107283 ( 0.258148 0.011801 1.020051)

28: 228.27 0.376874 ( -0.014467 0.613580 0.013579)

29: 255.66 3.919108 ( -1.621859 -0.070774 -1.132993)

30: 259.46 18.174754 ( -4.252942 -0.208486 0.209212)

31: 279.60 0.369691 ( -0.027769 0.607373 -0.004290)

32: 322.48 15.034383 ( -3.859017 -0.192369 0.324597)

33: 325.23 1.330563 ( 0.065542 -1.151418 -0.022451)

34: 327.08 1.909321 ( -1.281227 -0.044342 0.515570)

35: 345.63 14.405375 ( -0.152718 3.792145 0.041083)

36: 360.65 0.316738 ( -0.014442 -0.562606 -0.002089)

37: 372.29 1.791295 ( 1.310931 0.070921 -0.260239)

38: 390.38 2.001496 ( 1.374304 0.091388 0.323160)

39: 390.84 0.139012 ( -0.104986 0.357377 -0.016462)

40: 399.40 73.181336 ( -8.498539 -0.395525 -0.894275)

41: 416.33 0.005381 ( -0.033394 -0.065037 -0.005986)

42: 434.20 4.188136 ( -1.944061 -0.089642 -0.633031)

43: 446.78 0.010698 ( 0.011382 -0.102776 -0.002406)

44: 464.12 13.634891 ( 3.181305 0.163266 -1.867494)

45: 481.06 0.088536 ( -0.009057 0.297411 0.000235)

46: 508.35 55.939199 ( -7.462873 -0.348020 -0.351588)

47: 563.62 0.201728 ( -0.030333 0.448094 0.004400)

48: 576.06 7.205379 ( 2.661614 0.131414 -0.322363)

49: 581.39 0.108475 ( 0.052839 -0.323894 -0.027855)

50: 587.14 31.496218 ( 5.037947 0.285207 -2.456413)

51: 612.70 0.008390 ( 0.030723 -0.080298 0.031592)

52: 613.07 0.362838 ( -0.397112 -0.023038 -0.452337)

53: 632.30 12.198134 ( 0.169151 -3.488429 -0.019627)

54: 642.91 9.720788 ( -1.842021 -0.115854 2.512832)

55: 645.99 10.473498 ( 0.145743 -3.232848 -0.030878)

56: 661.02 8.702664 ( -2.900108 -0.130905 -0.524310)

57: 677.90 0.132471 ( -0.022063 -0.363288 -0.002513)

58: 682.04 14.225492 ( -1.108105 0.007621 -3.605210)

59: 691.77 61.396639 ( 7.660953 -0.855197 -1.405375)

60: 692.03 19.138503 ( 2.524544 3.537584 -0.500682)

61: 707.17 23.433387 ( 2.383830 -1.543614 -3.920204)

62: 707.30 17.399139 ( 0.935345 3.661392 -1.765921)

63: 719.91 0.845059 ( -0.493813 0.053245 -0.773546)

64: 720.62 30.023923 ( 0.249013 -5.472935 -0.094310)

65: 752.77 84.576083 ( 6.193727 0.200498 6.795118)

66: 754.37 0.156418 ( 0.179233 0.348729 -0.051785)

67: 758.58 41.962423 ( -4.744009 -0.184041 4.407145)

68: 760.69 2.187850 ( -0.205439 -1.439657 0.270244)

69: 773.51 6.678652 ( 0.201641 -2.575810 -0.056536)

70: 774.39 0.386494 ( 0.355866 0.299584 -0.412435)

71: 791.95 1.266091 ( 0.046293 -1.124245 -0.004529)

72: 807.99 12.148145 ( 3.236394 0.160595 -1.283787)

73: 828.28 0.058434 ( 0.038114 0.204213 -0.123604)

74: 828.38 0.081639 ( 0.061698 -0.263424 -0.091872)

75: 859.23 0.444235 ( -0.024587 0.666054 0.001453)

76: 864.93 2.101357 ( -1.070060 -0.078064 -0.974799)

77: 865.89 0.213757 ( -0.117503 0.440881 -0.074665)

78: 874.55 0.641771 ( -0.664758 -0.006975 -0.447012)

79: 890.97 3.067429 ( 0.630631 0.039289 -1.633460)

80: 892.69 6.772940 ( 0.113867 -2.599955 -0.014454)

81: 917.15 0.937062 ( -0.466329 -0.517350 -0.672271)

82: 917.33 2.688657 ( -0.988592 0.261729 -1.281734)

83: 920.79 0.039384 ( 0.076272 -0.062272 -0.172303)

84: 920.97 0.030707 ( -0.074846 -0.077697 0.138089)

85: 925.11 0.459002 ( 0.583611 -0.342106 -0.036920)

86: 925.37 0.463329 ( -0.531821 -0.421109 0.056235)

87: 933.84 3.396119 ( 1.131809 0.075763 1.452373)

88: 936.29 1.800216 ( 0.096087 -1.337882 0.032473)

89: 941.88 8.821515 ( 2.547182 0.115579 1.523160)

90: 960.36 43.152041 ( -0.302333 6.561612 0.076706)

91: 992.89 31.446361 ( 0.287044 -5.600128 -0.050314)

92: 996.46 1.494441 ( 1.041085 -0.002520 0.640762)

93: 996.82 0.211191 ( 0.011812 0.458436 0.029812)

94: 1004.24 38.086518 ( 5.928393 0.139202 1.709180)

95: 1026.96 4.869193 ( 1.639728 0.075439 1.474718)

96: 1028.31 2.548800 ( 0.089526 -1.593981 -0.002834)

97: 1032.30 144.558628 ( 0.489243 -12.011425 -0.211982)

98: 1042.38 3.739364 ( 1.764897 0.375648 -0.695265)

99: 1042.47 10.347687 ( -3.000925 -0.012500 1.158439)

100: 1058.11 3.118539 ( -0.847071 -0.001806 -1.549518)

101: 1063.41 3.792784 ( 0.053575 -1.946619 -0.024286)

102: 1073.09 8.864802 ( 0.317092 -0.085749 2.959206)

103: 1073.65 1.440257 ( -0.018523 1.182252 0.205412)

104: 1093.44 16.150495 ( 2.971158 0.298873 -2.689497)

105: 1093.45 1.126580 ( -0.510375 0.812094 0.454532)

106: 1132.43 16.908455 ( -3.188857 -0.229988 2.585876)

107: 1136.52 12.610153 ( -0.175429 3.546353 0.052552)

108: 1153.69 0.001950 ( -0.005322 -0.043639 0.004201)

109: 1153.69 0.000594 ( -0.017424 0.014980 0.008155)

110: 1157.09 0.836214 ( 0.497892 0.403154 -0.652521)

111: 1157.13 1.873898 ( 0.204593 -1.338988 -0.197868)

112: 1167.93 0.664238 ( -0.640656 -0.055017 0.500770)

113: 1170.10 1.127498 ( 0.000658 -1.061434 -0.029230)

114: 1170.96 1.954824 ( 1.277681 0.009763 0.567680)

115: 1172.76 0.059095 ( 0.022884 -0.242010 0.001516)

116: 1184.27 19.896397 ( 4.270523 0.195788 1.273065)

117: 1251.19 0.945150 ( -0.038017 0.971370 -0.012074)

118: 1258.96 1.526780 ( -0.353964 1.157348 0.249069)

119: 1260.02 32.281252 ( -4.481868 -0.329490 3.476427)

120: 1278.04 130.111214 ( -0.442644 11.396875 -0.162840)

121: 1281.43 28.261550 ( -1.769088 0.552477 4.982635)

122: 1300.11 39.116440 ( -0.252382 6.249171 0.024640)

123: 1302.05 0.896125 ( 0.470564 0.160342 0.805596)

124: 1303.19 0.083346 ( -0.043339 0.279774 -0.056521)

125: 1320.77 277.709922 ( 13.016969 0.754806 -10.377799)

126: 1332.09 3.036177 ( 0.100419 -1.739510 -0.014036)

127: 1332.39 70.768100 ( -7.645344 -0.432698 3.482756)

128: 1332.84 3.801308 ( -1.876344 -0.050505 0.527343)

129: 1333.04 53.357107 ( 7.077655 0.380489 -1.766108)

130: 1336.38 42.729521 ( -5.668405 -0.315070 3.240283)

131: 1338.52 5.756877 ( 0.237354 -2.386936 -0.055484)

132: 1404.79 2.012862 ( 0.041767 -1.418041 -0.016616)

133: 1411.43 25.472431 ( -4.576674 -0.187004 -2.119320)

134: 1430.77 6.723989 ( -2.156107 -0.099296 -1.437126)

135: 1434.02 1.429057 ( -0.032743 -0.414656 1.120734)

136: 1434.08 5.499614 ( 0.072498 0.047063 -2.343532)

137: 1434.12 5.283281 ( -0.096193 2.283384 0.245331)

138: 1445.90 53.589324 ( -7.139541 -0.331776 1.583098)

139: 1461.85 12.343203 ( -0.098692 3.511541 0.050462)

140: 1471.13 4.048612 ( -0.070084 2.010604 0.034228)

141: 1475.58 14.635496 ( 2.856090 0.185151 -2.538497)

142: 1482.15 0.988959 ( -0.025314 -0.994107 -0.008371)

143: 1482.34 6.332238 ( 2.501031 0.086489 0.263818)

144: 1561.78 5.500268 ( -0.343099 -2.308541 0.230627)

145: 1562.18 14.626731 ( -3.307224 0.139691 1.915590)

146: 1573.11 2.442196 ( 0.136398 -0.213786 -1.542040)

147: 1573.12 0.738340 ( 0.037000 0.657434 -0.552043)

148: 1589.71 0.117036 ( -0.004755 -0.340908 -0.028186)

149: 1591.87 4.188244 ( 1.482449 0.069012 1.409193)

150: 1596.26 2.512657 ( 1.420356 0.098595 0.696796)

151: 1596.31 0.368846 ( -0.096682 0.598821 -0.030202)

152: 1609.07 15.173821 ( 0.169960 -3.891307 -0.051640)

153: 1618.98 149.761991 (-11.981116 -0.572295 -2.426381)

154: 1619.60 9.072676 ( -2.956571 0.005681 -0.575611)

155: 1657.31 722.876391 (-19.595966 -0.641195 -18.397374)

156: 2527.10 10.797552 ( -0.977712 3.135721 0.094246)

157: 2678.14 5644.066877 ( 74.913223 3.706899 -4.281913)

158: 2929.11 9.661929 ( 0.905294 -0.180608 -2.968123)

159: 2934.46 30.921078 ( -0.235584 5.555305 -0.064502)

160: 2973.24 23.879794 ( 0.204885 -4.882295 0.031832)

161: 2976.43 3.820008 ( 0.908031 -0.114572 -1.726952)

162: 3095.35 2.112960 ( 0.708989 -0.706620 1.054032)

163: 3095.37 2.214811 ( -0.304794 -1.329298 -0.595718)

164: 3100.54 0.126744 ( 0.197512 -0.248656 -0.160946)

165: 3100.55 0.129453 ( -0.153103 -0.290547 0.146951)

166: 3102.67 0.778761 ( -0.875903 -0.043974 -0.098085)

167: 3102.67 0.132047 ( -0.028235 0.362132 0.010502)

168: 3108.59 6.450571 ( 0.843792 -1.052729 2.151824)

169: 3108.60 4.967162 ( 0.480018 1.537349 1.540553)

170: 3112.64 5.023311 ( 2.205402 0.095944 -0.387695)

171: 3112.65 3.279606 ( -1.781362 -0.141274 0.293932)

172: 3115.78 21.656382 ( -0.134057 -0.782235 4.585468)

173: 3115.79 12.419090 ( 0.166334 -3.364647 -1.034686)

174: 3121.16 6.259625 ( 0.702343 -2.239342 0.866999)

175: 3121.17 11.398246 ( 0.203535 3.303224 0.667480)

176: 3124.95 13.613995 ( -2.268874 -0.152865 -2.905656)

177: 3124.98 19.708916 ( -0.251624 4.432332 -0.006076)

178: 3128.10 28.884243 ( -5.175650 -0.716864 1.258172)

179: 3128.15 4.969484 ( -1.359029 1.738889 0.314307)

**
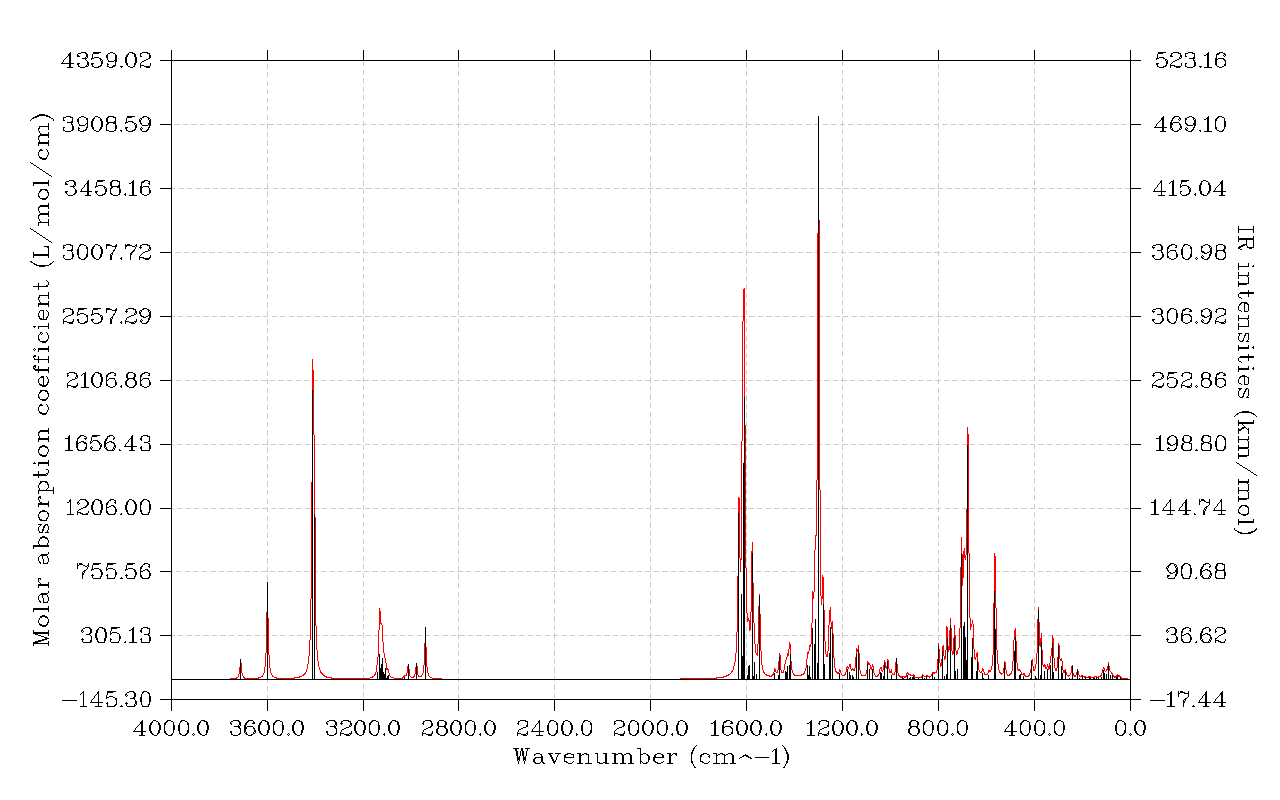
**

**Figure S1**: IR spectrum (unscaled frequencies) for $\left[ Co\left( EDA2BB \right)\left( OH_{2} \right)_{2} \right]$ complex in gas phase

**
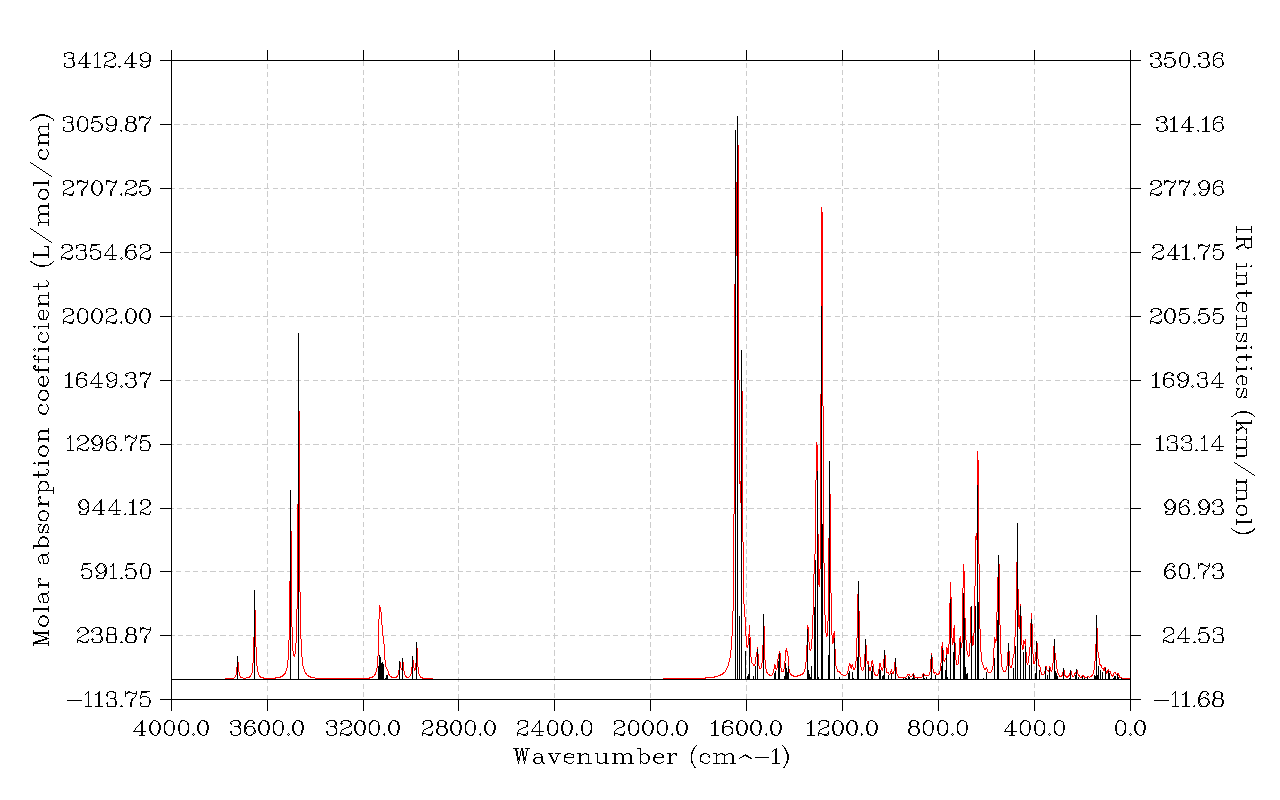
**

**Figure S2**: IR spectrum (unscaled frequencies) for $\left[ Ni\left( EDA2BB \right)\left( OH_{2} \right)_{2} \right]$ complex in gas phase

**
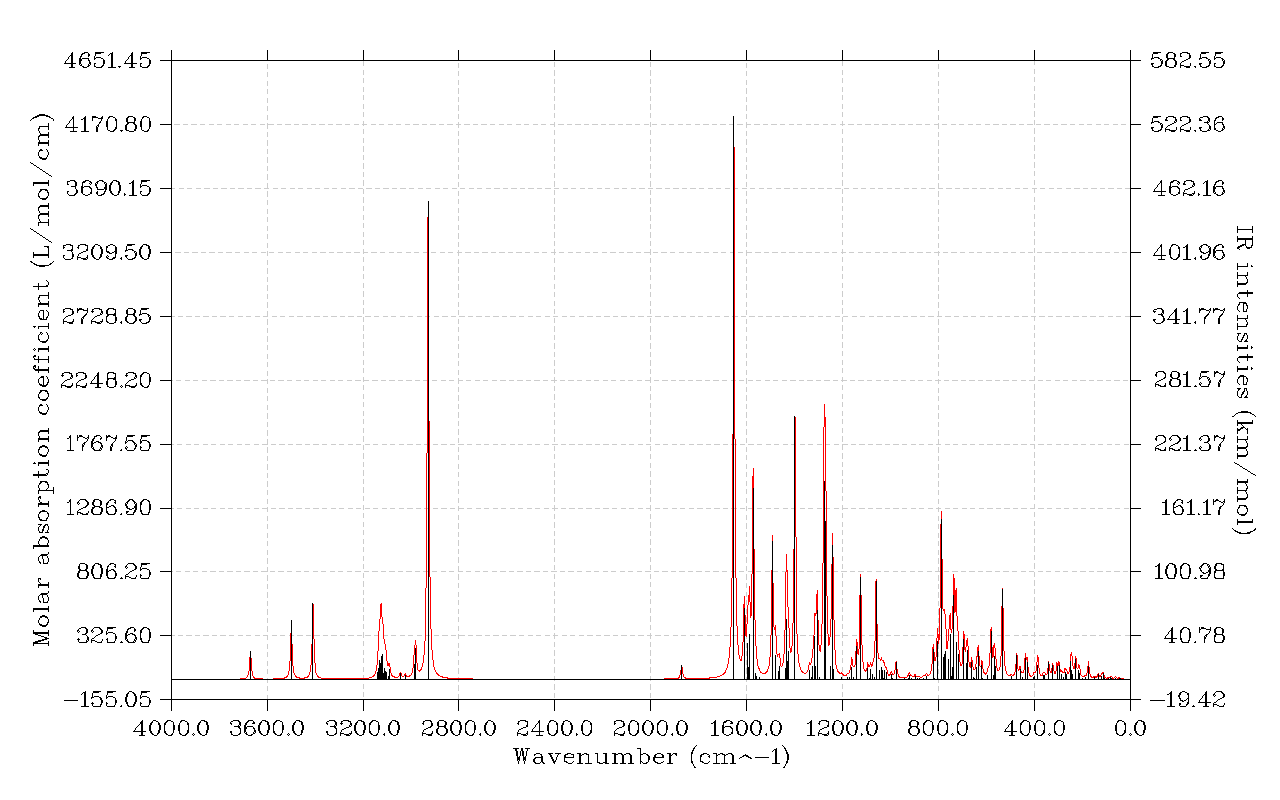
**

**Figure S3**: IR spectrum (unscaled frequencies) for $\left[ Mn\left( EDA2BB \right)\left( OH_{2} \right)_{2} \right]$ complex in gas phase

**
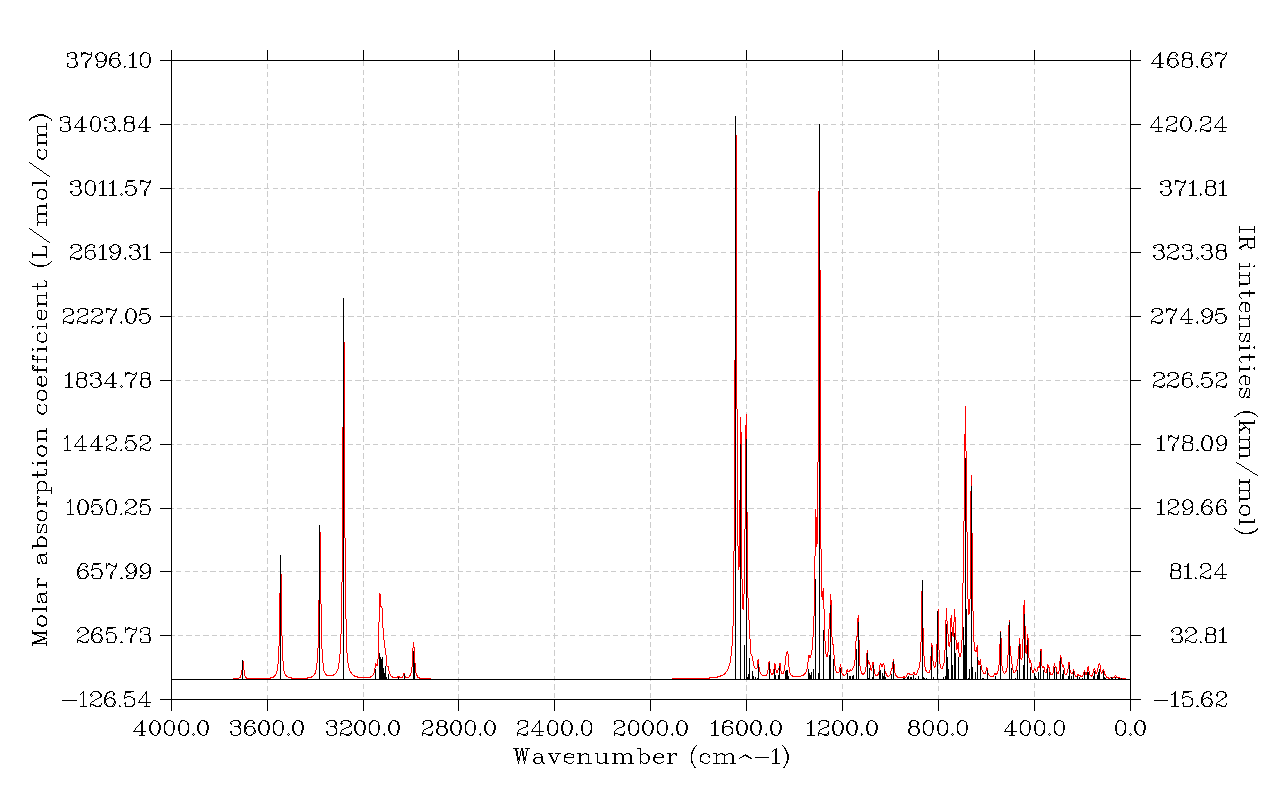
**

**Figure S4** IR spectrum (unscaled frequencies) for $\left[ Co\left( EDA2BB \right)\left( OH_{2} \right)_{2} \right]$ complex in gas phase

**
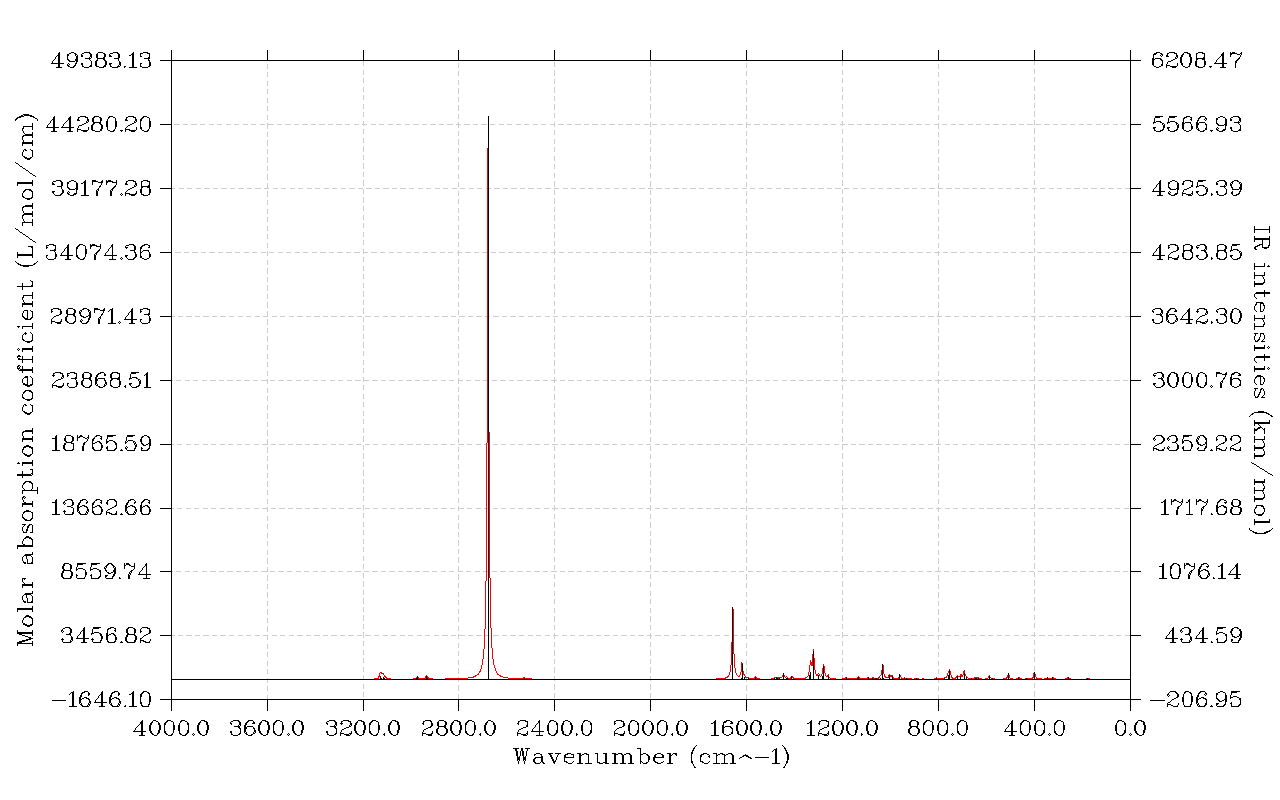
**

**Figure S5**: IR spectrum (unscaled frequencies) for $\boldsymbol{EDA}\mathbf{2}\boldsymbol{BB}$ ligand in gas phase
